# Evaluation of gliovascular functions of AQP4 readthrough isoforms

**DOI:** 10.3389/fncel.2023.1272391

**Published:** 2023-11-23

**Authors:** Shayna M. Mueller, Kelli McFarland White, Stuart B. Fass, Siyu Chen, Zhan Shi, Xia Ge, John A. Engelbach, Seana H. Gaines, Annie R. Bice, Michael J. Vasek, Joel R. Garbow, Joseph P. Culver, Zila Martinez-Lozada, Martine Cohen-Salmon, Joseph D. Dougherty, Darshan Sapkota

**Affiliations:** ^1^Department of Genetics, Washington University School of Medicine, Saint Louis, MO, United States; ^2^Department of Psychiatry, Washington University School of Medicine, Saint Louis, MO, United States; ^3^Department of Radiology, Washington University School of Medicine, Saint Louis, MO, United States; ^4^Department of Biological Sciences, University of Texas at Dallas, Richardson, TX, United States; ^5^Intellectual and Developmental Disabilities Research Center, Washington University School of Medicine, Saint Louis, MO, United States; ^6^Department of Physics, Washington University in St. Louis, Saint Louis, MO, United States; ^7^Department of Biomedical Engineering, Washington University in St. Louis, Saint Louis, MO, United States; ^8^Department of Neuroscience, Washington University School of Medicine, Saint Louis, MO, United States; ^9^Imaging Science PhD Program, Washington University in St. Louis, Saint Louis, MO, United States; ^10^Department of Pediatrics, The Children's Hospital of Philadelphia, Philadelphia, PA, United States; ^11^Center for Interdisciplinary Research in Biology (CIRB), Collège de France, The National Centre for Scientific Research (CNRS), National Institute of Health and Medical Research (INSERM), Université PSL, Paris, France; ^12^Department of Neuroscience, University of Texas at Dallas, Richardson, TX, United States

**Keywords:** AQP4, AQP4x, astrocyte, blood-brain barrier, glymphatic, readthrough

## Abstract

Aquaporin-4 (AQP4) is a water channel protein that links the astrocytic endfeet to the blood-brain barrier (BBB) and regulates water and potassium homeostasis in the brain, as well as the glymphatic clearance of waste products that would otherwise potentiate neurological diseases. Recently, translational readthrough was shown to generate a C-terminally extended variant of AQP4, known as AQP4x, which preferentially localizes around the BBB through interaction with the scaffolding protein α-syntrophin, and loss of AQP4x disrupts waste clearance from the brain. To investigate the function of AQP4x, we generated a novel AQP4 mouse line (AllX) to increase relative levels of the readthrough variant above the ~15% of AQP4 in the brain of wild-type (WT) mice. We validated the line and assessed characteristics that are affected by the presence of AQP4x, including AQP4 and α-syntrophin localization, integrity of the BBB, and neurovascular coupling. We compared AllX^Hom^ and AllX^Het^ mice to WT and to previously characterized AQP4 NoX^Het^ and NoX^Hom^ mice, which cannot produce AQP4x. An increased dose of AQP4x enhanced perivascular localization of α-syntrophin and AQP4, while total protein expression of the two was unchanged. However, at 100% readthrough, AQP4x localization and the formation of higher order complexes were disrupted. Electron microscopy showed that overall blood vessel morphology was unchanged except for an increased proportion of endothelial cells with budding vesicles in NoX^Hom^ mice, which may correspond to a leakier BBB or altered efflux that was identified in NoX mice using MRI. These data demonstrate that AQP4x plays a small but measurable role in maintaining BBB integrity as well as recruiting structural and functional support proteins to the blood vessel. This also establishes a new set of genetic tools for quantitatively modulating AQP4x levels.

## Graphical Abstract

*Aqp4* readthrough determines the perivascular concentrations of AQP4 and a-Syn. 
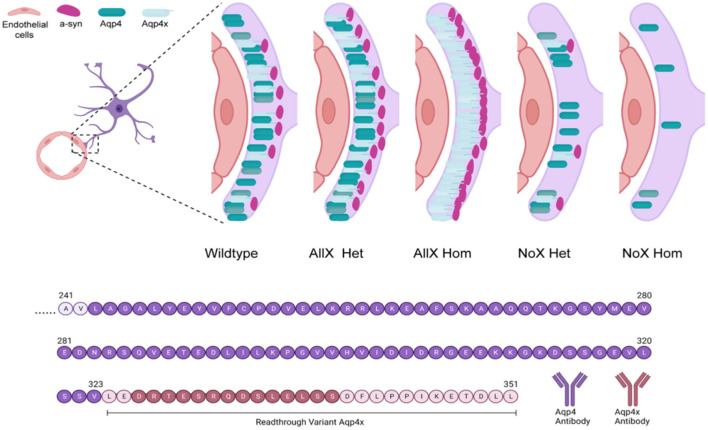

## Introduction

Aquaporin-4 (AQP4) is a transmembrane water channel protein that is expressed by astrocytes and is highly localized to their endfeet processes, which surround the CNS vasculature. This channel facilitates the clearance of extracellular solutes and other waste products, such as amyloid beta, from the interstitial fluid via a system that has been termed the “glymphatic system” (Iliff et al., 2012; Jessen et al., 2015; Mestre et al., 2018; Benveniste et al., 2019). Indeed, mice lacking AQP4 have a reduced rate of interstitial solute clearance by up to 70% (Iliff et al., 2012).

Via a process of alternative translation, several isoforms of the AQP4 protein are made from the same transcript and may serve distinct roles in the brain. Specifically, the *Aqp4* transcript can initiate translation at two different in-frame start sites that form the M1 or the M23 isoform, respectively. Additionally, the transcript undergoes stop-codon readthrough in mice, rats, and humans, which produces a protein with a C-terminal extension containing 29 amino acids before terminating at a second stop codon. In the mouse brain, 10–15% of the AQP4 protein is estimated to contain the C-terminal readthrough extension (De Bellis et al., 2017; Palazzo et al., 2019; Sapkota et al., 2019). Having two start sites and two termination sites brings the total number of AQP4 isoforms to 4: M1, M23, M1x, and M23x, with the alternative initiation and readthrough being independent events (De Bellis et al., 2017). In two independent studies, the ability of the *Aqp4* transcript to produce a readthrough variant was genetically abolished through additional stop codons, generating AQP4 “NoX” mice (De Bellis et al., 2017; Sapkota et al., 2022). In these mice, AQP4 was no longer preferentially localized to the endfoot compartment, but total AQP4 levels remained unchanged (De Bellis et al., 2017; Sapkota et al., 2022). Loss specifically of this endfoot localization corresponded to deficits in amyloid beta clearance, while drugs enhancing readthrough promoted clearance (Sapkota et al., 2022).

Some details are known regarding the mechanism of AQP4 localization to the endfeet. The dystrophin-associated protein complex recruits and anchors proteins to the cell membrane. One member of this complex, α-syntrophin, binds to other membrane-associated proteins using its PDZ domain to recruit or stabilize them to the plasma membrane (Constantin, 2014). Mice that lack the dystrophin Dp71 show a 70% reduction in polarized perivascular AQP4 as well as a complete loss of α-syntrophin expression (Belmaati Cherkaoui et al., 2021). In α-syntrophin knockout (KO) mice, polarization of AQP4 toward the perivascular astrocytic membrane is significantly reduced without changing overall AQP4 protein levels, suggesting that α-syntrophin anchors or localizes AQP4 to the perivascular membrane (Amiry-Moghaddam et al., 2003; Mestre et al., 2018). These results were supported by earlier data demonstrating that α-syntrophin KO mice had astrocyte processes change their orientation from facing the blood vessel to facing the neuropil (Neely et al., 2001). Therefore, the later discovery of AQP4x led to the hypothesis that the C-terminal extension enables binding to α-syntrophin and thus localization to the neurovasculature. Indeed, α-syntrophin co-immunoprecipitation, and immunofluorescence comparing AQP4 to AQP4x-transfected cells revealed α-syntrophin interacts specifically with AQP4x *in vitro* (De Bellis et al., 2017). Furthermore, NoX mice, which have no AQP4x, showed a 40% reduction in α-syntrophin total protein expression compared to wild type (WT), as well as qualitatively less perivascular localization of α-syntrophin (Palazzo et al., 2019). This suggests that AQP4x loss reduces α-syntrophin localization and that α-syntrophin loss reduces AQP4x localization. However, it is unknown how intermediate levels of AQP4x quantitatively regulate the localization of both proteins or whether increases in AQP4x production might further recruit α-syntrophin to the perivascular space.

Research into how AQP4 isoforms function is further complicated by the fact that they are known to form tetramers (including heterotetramers harboring multiple AQP4 isoforms) and superstructures containing “Orthogonal Arrays of Particles” (OAPs) that are comprised of multiple tetramers aligned in close proximity (Nico et al., 2001; Crane and Verkman, 2009; Liebner et al., 2011; Zhu et al., 2022). While OAP size can be decreased by the presence of M1, the isoform is not required for the arrangement, yet mice missing the M23 isoform lose the ability to form OAPs and show disrupted endfoot localization (Nicchia et al., 2008; de Bellis et al., 2021). Thus, both the presence of readthrough isoforms and the ability to form OAPs can influence AQP4 endfoot localization. Overexpression of individual isoforms in culture (De Bellis et al., 2017) or examination of NoX brain lysates (Palazzo et al., 2019) revealed that the size of each OAP changed due to the change in weight of their constituent components; however, there are still many unknowns regarding how AQP4x affects OAP formation *in vivo* and the functional consequences of modulating the relative levels of AQP4x and OAP formation.

Finally, the integrity of the BBB is reliant on the cohesive functionality of its components, such as astrocyte endfeet, endothelial cells (ECs), and pericytes, but it is unclear if AQP4 readthrough affects BBB function. A prior study in global KOs suggested that AQP4 deletion disrupted endfoot morphology (Zhou et al., 2008), but this did not replicate in all studies (Saadoun et al., 2009). While NoX mice had a visually normal BBB ultrastructure (Palazzo et al., 2020), this was not quantitatively evaluated, and it is unclear if upregulating readthrough may have an impact. Furthermore, maturation of the BBB and AQP4 have been observed to coincide with each other (Nico et al., 2001; Nicchia et al., 2004), and astrocyte co-culture with ECs (bEnd3) enhanced AQP4 polarization specifically toward processes that were in contact with the ECs (Nicchia et al., 2004). This suggests ECs may promote AQP4 readthrough or localization of the AQP4x isoform. Thus, while global KOs have normal BBB permeability to large molecules but decreased water permeability at baseline (Papadopoulos and Verkman, 2005; Haj-Yasein et al., 2011), it is unclear if specifically altering AQP4x levels might influence BBB structure, integrity, or function, or if such alterations impact key neurovascular unit functions such as regulating the coupling between neuronal activity and blood flow in the brain.

To enable careful studies of AQP4x in these roles, we generated and validated a genetic series of mice modulating AQP4 readthrough from 0% (NoX^Hom^ mice, homozygous for the NoX *Aqp4* allele) to 100% with novel AQP4 AllX^Hom^ mice. We then deeply characterized the consequences of systematically modulating AQP4x levels on protein localization, structure and function of the endfoot, and the influx or efflux of MR contrast agents. From these studies, we define a role for AQP4x in influx/efflux in the brain and a quantitative influence on α-syntrophin localization. Overall, this establishes a genetic toolset for quantitatively modulating the AQP4 readthrough levels for studies in health and disease.

## Results

### Generation and validation of an AQP4 obligatory readthrough mutant (AllX)

**Figure 1:**
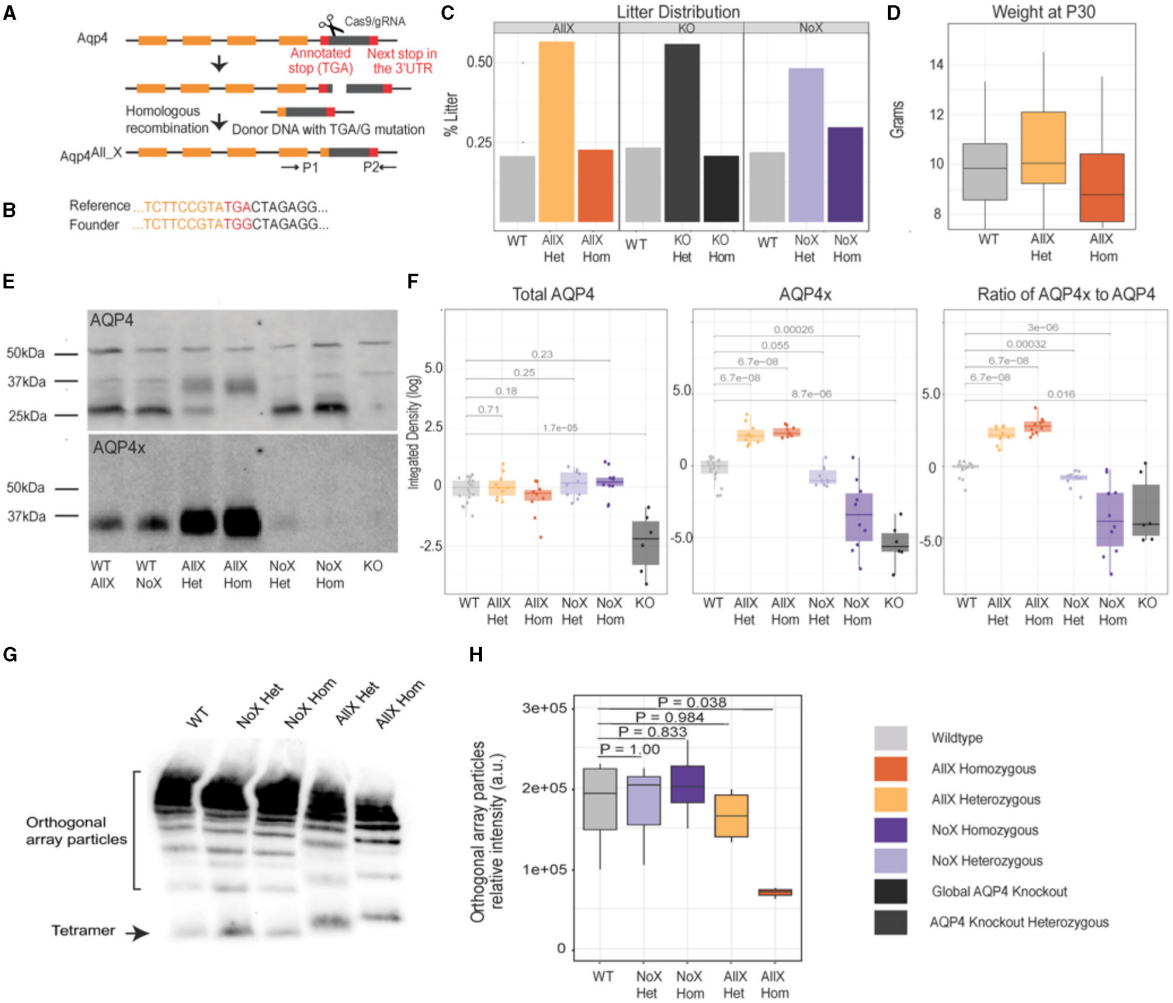
Generation of an obligate AQP4 readthrough line and characterization of AQP4x production. **(A)** CRISPR/Cas9 construction of the AllX mutant mouse line. **(B)** Reference and founder sequence showing conversion of the TGA stop codon to a TGG (encoding tryptophan). **(C)** Litter distribution among the genotypes follows the Mendelian ratio. n (litters) = 13 Aqp4 (KO) line, 36 AllX line, and 32 NoX line. **(D)** Weight distribution at P30 between AllX and WT mice, *n* = 10 WT, 9 AllX^Hom^, 22 AllX^Het^. **(E)** Western blot showing AQP4 and AQP4x expression, *n* = 3 per genotype, 1 global AQP4 KO mouse as a specificity control for the antibody. Total brain lysates probed with anti-AQP4 and anti-AQP4x antibodies show a significant increase in AQP4x compared to AQP4 in the AllX variants. AQP4 expression is enhanced in the no-readthrough variants and decreases in the AllX variants. The anti-AQP4 antibody exhibits two non-specific bands at 40 and 50 kDa, as evidenced by their presence in the global AQP4 knockouts (asterisk). **(F)** Quantification of Western blot as box plots (log10 scale) of AQP4 and AQP4x total and the relative ratio of AQP4x to AQP4 quantifying Western blot using ImageJ, normalized to 50 kDa band and wildtype. WT AllX and WT NoX merged and analyzed using the Wilcox test. **(G)** BN-PAGE with anti-AQP4 antibody showing orthogonal array particles and tetramer expression. **(H)** Quantification of orthogonal array particle signal from **(G)**. *n* = 3–4 mice per genotype. One-way ANOVA, with *post-hoc P*-values from Dunnett's test.

To date, there is no gain-of-function mouse model to increase AQP4-mediated bulk flow and potentially “glymphatic” clearance, as there is no genetic method to increase the perivascular pool of AQP4. We and others have recently shown that this pool of AQP4 contains readthrough-generated AQP4x (De Bellis et al., 2017; Sapkota et al., 2022). We therefore hypothesized that promoting readthrough should upregulate the expression of AQP4x and thus perivascular localization. Normally, approximately 15% of translating ribosomes read past the *Aqp4* stop codon to generate AQP4x in the rodent brain (De Bellis et al., 2017; Palazzo et al., 2019; Sapkota et al., 2019). To generate a mouse with 100% readthrough, we used CRISPR-Cas9 to precisely mutate the stop codon to a sense codon (TGA->TGG) (Figures 1A, B). Genotype distribution among the litters followed the normal Mendelian ratio, and weight distribution within AllX genotypes and WT was not significantly different, although the homozygotes tended to be slightly smaller (Figures 1C, D). Mouse behavior appeared phenotypically normal, at least within what was observable during the course of normal handling for breeding and husbandry.

Immunoblot of whole brain tissue with an anti-AQP4x antibody showed that this mouse (AQP4 “AllX”) indeed expresses increased levels of AQP4x protein in the expected “WT <AllX” order (Figures 1E, F, Supplementary Figure S1). There is not a significant difference between AQP4x expression within the AllX^Het^ and AllX^Hom^ genotypes (*p* = 0.35). In addition, with an increase in AQP4x production, the level of normal-length AQP4 protein should diminish. Using an anti-AQP4 antibody that recognizes AQP4x as well as the normal-length AQP4, we observed changes in isoform abundance consistent with increased stop codon readthrough in AllX mutant lines (Figures 1E, F). These immunoblot experiments also include our previously described AQP4 NoX mice (where readthrough is abolished with extra stop codons) for comparison and AQP4 KO (global KO) mice as negative controls. As expected, loss of AQP4x is observed in NoX mice at the expected rates, and all AQP4 isoforms were absent in the global KO. These results confirm that AllX mice faithfully overexpress the readthrough-extended variant of AQP4 and establish an allelic series to modulate relative AQP4x levels.

The additional specific bands seen on the AQP4 Western blot can be explained by the M1 and M23 alternative initiation events. Specifically, WT littermates from both the AllX and NoX lines show a faint band at 34 kDa indicating the expression of AQP4 M1 isoform that contains an additional 22 residues at its N-terminus, as seen in Figure 1E (Crane and Verkman, 2009). A strong band is seen at 32 kDa which is AQP4′s M23 isoform. The stronger expression of the M23 band is consistent with previous findings that M23 is three to ten times more abundant than M1 (Neely et al., 1999). Furthermore, bands at 35 and 38 kDa are identified as the readthrough isoforms M23x and M1x, respectively. The AllX mutants show a decreased expression of AQP4 M23, with a complete loss in the AllX^Hom^ and a slight increase in band density at 34 kDa. This is consistent with expectations since the AQP4 antibody detects all isoforms. With AQP4x-specific antibodies, there is a significant increase in AQP4x expression in the complete readthrough variants compared to WT (Figure 1F). Slight AQP4x expression is still seen in the NoX^Het^ mice, as expected.

*In vivo*, functional AQP4 does not exist as a monomeric protein but instead forms tetramers, which, in turn, assemble into supramolecular complexes known as orthogonal array particles (OAPs). Since AQP4 initiation site variants are known to have different propensities to assemble into OAP particles (M23 > M1), we asked if the process is also influenced by the readthrough variant. In Blue Native Polyacrylamide Gel Electrophoresis (BN-PAGE), which allows for the separation of protein complexes in native states, we observed that OAP assemblies remain in normal abundance in NoX^Het^, NoX^Hom^, and AllX^Het^ mice but are significantly reduced in abundance in AllX^Hom^ mice when compared to WT mice (Figures 1G, H), along with a subtle shift in size corresponding to the increased size of AQP4x subunits. This suggests that neither AQP4x nor unextended AQP4 is an absolute requirement for the formation of OAPs, and the two may in fact cooperate in the process. However, when alone, AQP4x is less efficient in assembling into OAPs.

### AQP4 NoX and AllX lines show gene-dose dependent pattern of endfoot localization for AQP4, AQP4x, and α-syntrophin

α-syntrophin is a scaffolding protein that acts as an adaptor between AQP4 and the dystrophin complex via AQP4′s C-terminal domain (Constantin, 2014). Though AQP4′s expression level is not dependent on α-syntrophin, its endfoot localization is disrupted in α-syntrophin KO mice (Neely et al., 2001). Furthermore, previous studies have shown that the AQP4x variant colocalizes with α-syntrophin and is more enriched by co-immunoprecipitation than other AQP4 isoforms in transfected cells. Here, we assessed how raising and lowering relative AQP4x levels impacted its perivascular localization and whether such modulation of AQP4x could also alter α-syntrophin localization.

**Figure 2:**
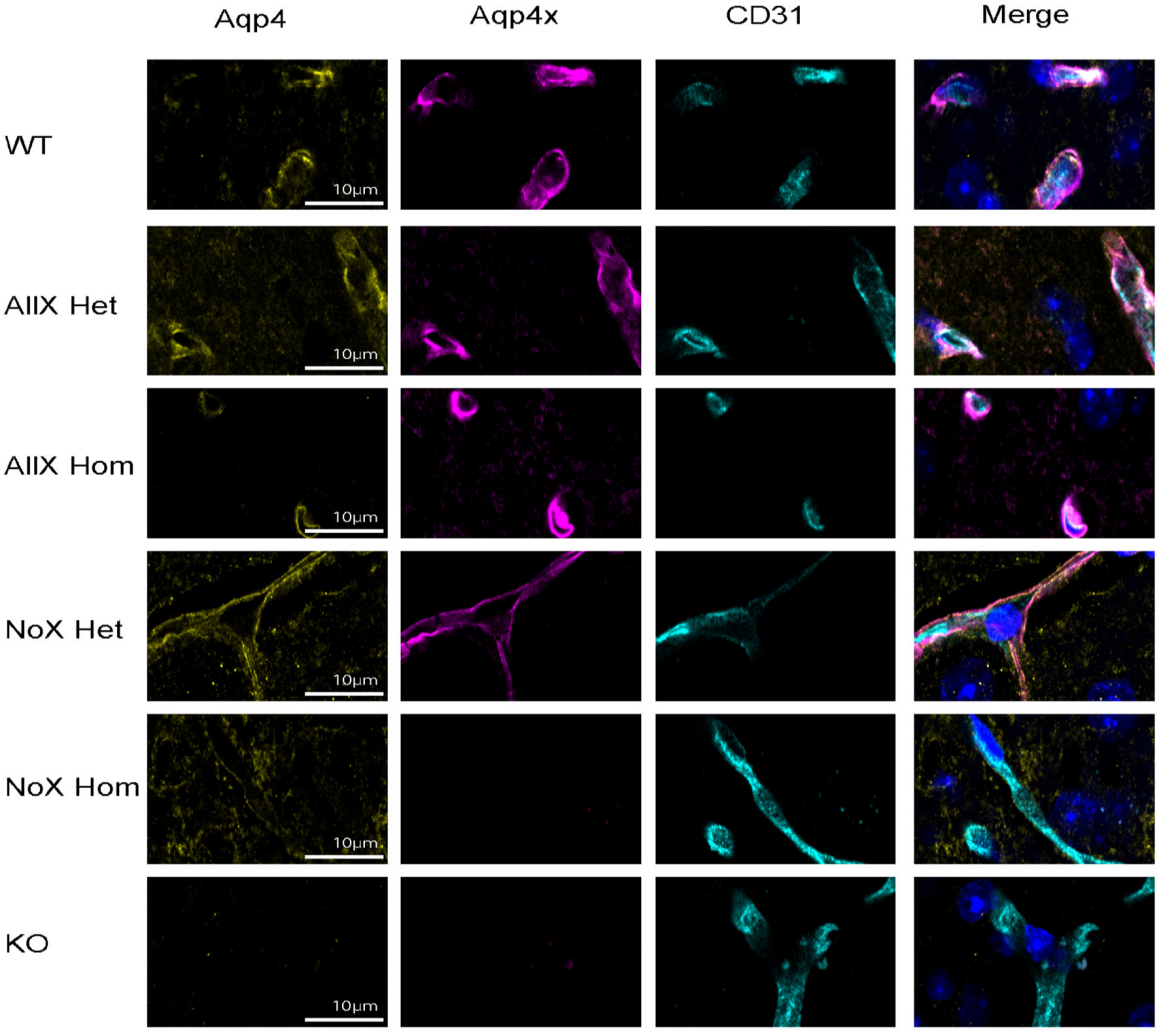
AQP4 is enhanced at the blood vessel when AQP4x is upregulated. Immunofluorescence staining of AQP4, AQP4x, and CD31 among the genotypes at the blood vessel (CD31) in the cortex. Note that the AQP4 and AQP4x signal decreases in the NoX variant at the blood vessel and increases in the AllX variant, *n* = 5 mice per genotype.

**Figure 3:**
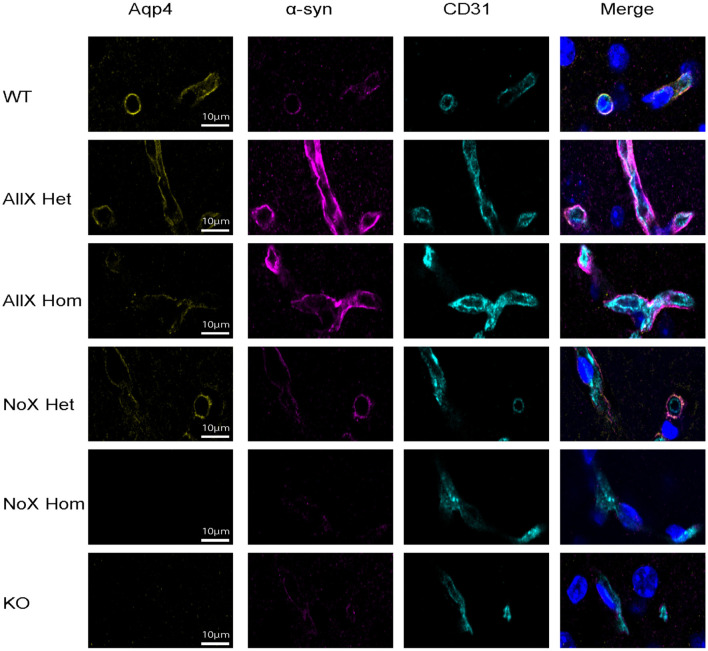
α-syntrophin is enhanced at the blood vessel when AQP4x is upregulated. Immunofluorescence staining of AQP4, α-syntrophin, and CD31 among the genotypes at the blood vessel (CD31) in the cortex. Note that the AQP4 and α-syntrophin signal decreases in the NoX variant at the blood vessel and increases in the AllX variant, *n* = 5 mice per genotype.

**Figure 4:**
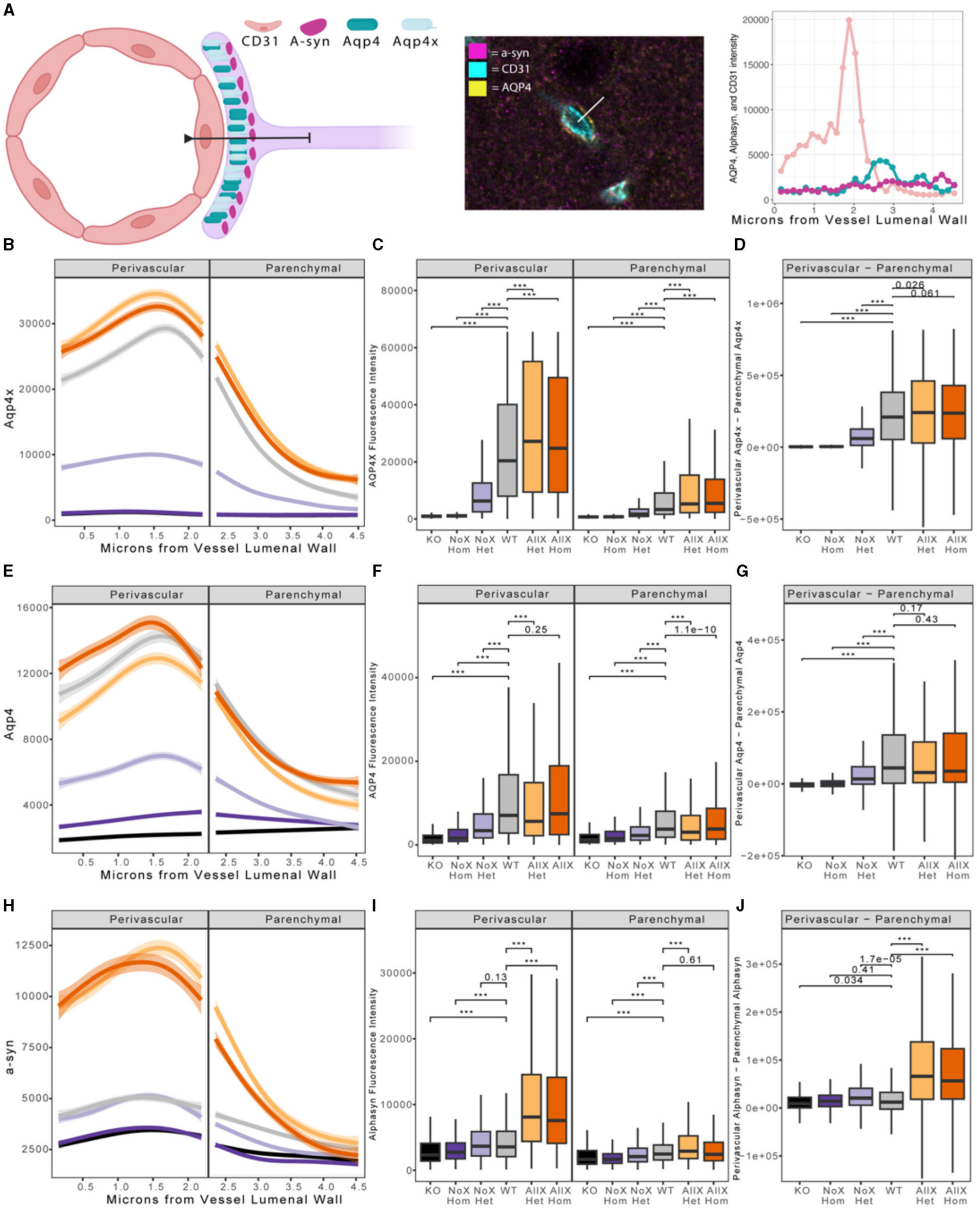
Blood vessel localization of AQP4X, AQP4, and α-syntrophin is altered in AQP4x mutants. **(A)** Depiction of astrocyte endfoot contacting the blood vessel wall (CD31). α-syntrophin is anchored to AQP4 via the C-terminus of AQP4x in the endfoot. Quantification of endfoot localization was performed along a line extending from the vessel luminal wall out at least 4.5 μm into parenchymal space. **(B, E, H)** Loess curve fit of fluorescent intensities of AQP4x **(B)**, AQP4 **(E)** α-syntrophin **(H)**, and along a line from the vessel luminal wall toward the parenchyma as diagrammed in **(A)** for each genotype shown. Shading depicts the edges of the 95% confidence interval. Significance testing with linear mixed models reveals a significant effect of genotype in AQP4x, α-syntrophin, and AQP4 channels (*p*-values = 1.862e-9, 2.049e-09, and 2.128e-10, respectively). Modeling also indicated a highly significant interaction effect of genotype and distance for the three channels AQP4x, α-syntrophin, and AQP4 (*p*-values = 2.2e-16, 2.2e-16, and 2.2e-16, respectively). **(C, F, I)** Boxplot depicting AQP4x levels shows significant changes across the genotypes in both the perivascular and parenchymal regions for AQP4x, a-syn, and AQP4, respectively. *Post-hoc p*-values generated from the Wilcox test are shown, with *** representing a *p*-value of 2.2e-16. **(D, G, J)** Boxplot of perivascular intensity of a given line minus parenchymal intensity of a given line. *Post-hoc p*-values generated from the Wilcox test highlight significant changes in localization, with *** representing a *p*-value of 2.2e-16. Color code as in Figure 1.

As AQP4x is known to highly localize to astrocyte endfeet (De Bellis et al., 2017; Sapkota et al., 2019), we utilized immunofluorescence to measure expression and endfoot localization of AQP4, AQP4x, and α-syntrophin in the NoX, AllX, and WT allelic series, including global AQP4 KOs as staining controls (Figures 2, 3). To quantify endfoot localization, we manually drew a line of at least 4.5 μm starting from the vessel lumenal wall and extending out toward the parenchyma and plotted the histogram of pixel intensities, divided into perivascular (<2.25 μm from the lumenal wall) and parenchymal (>2.25 μm from the lumenal wall), for each channel (Figures 4A, B). AQP4x perivascular and parenchymal levels increased with AQP4x gene dose, with NoX^Hom^ exhibiting no expression, NoX^Het^ significantly less than WT, and both AllX mutants significantly greater than WT (Figures 4B, C). Our initial round of quantification showed no difference between levels of AQP4X comparing the AllX^Hom^ and AllX^Het^, but we have determined that this was at least partially due to pixel saturation during this round of imaging where it was tough to find a dynamic range that could image all genotypes. A subsequent re-imaging of just AllX^Hom^ and AllX^Het^ animals in non-saturating settings did detect a small but significant increase, showing AllX^Hom^ having ~10% more perivascular AQP4x protein than AllX^Hets^ (Supplementary Figure 2).

As a measure of perivascular localization, we subtracted the summation of parenchymal intensities from the summation of perivascular intensities and found significant deficits in localization for the NoX^Hom^, NoX^Het^, and KO lines compared to WT, while AllX^Hets^ were significantly more localized and AllX^Homs^ were trending toward perivascular localization compared to WT animals (Figure 4D). Though total brain AQP4 protein levels were similar between WT and the AQP4x mutants (Figure 1F), perivascular and parenchymal expression of AQP4 was decreased (compared to WT) in NoX^Homs^ and NoX^Hets^, while AllX^Hets^ showed slight decreases and AllX^Homs^ showed no difference in perivascular and a slight increase in parenchymal levels (Figures 4E, F). Perivascular localization (perivascular-parenchymal) was decreased in NoX mutants but remained at similar levels between WT and AllX variants (Figure 4G), consistent with a minimal level of AQP4x being required for localizing AQP4 to the astrocyte endfoot. Interestingly, α-syntrophin perivascular expression is very sensitive to the amount of AQP4x present, with NoX^Hom^ showing a significant decrease and AllX^Het^ and AllX^Hom^ exhibiting a robust increase in perivascular signal (Figures 4H, I). Furthermore, localization (perivascular-parenchymal) of α-syntrophin was also significantly increased in AllX^Het^ and AllX^Hom^ mice (Figure 4J). Thus, while α-syntrophin total brain protein expression was not significantly affected by genotype (Supplementary Figure 3), increasing readthrough can increase recruitment of α-syntrophin to the blood vessels, even as total AQP4 at this location remains unchanged. This indicates that not only is α-syntrophin required to localize AQP4 to endfeet, but the amount of readthrough of AQP4 also plays a quantitative role in the localization of α-syntrophin. A full statistical analysis of immunofluorescence data can be found in Supplementary Table 1.

### Neurovascular coupling

A key function of the neurovascular unit, composed of mural cells, ECs, and the AQP4x-containing astrocyte endfoot, is the upregulation of blood flow to brain regions undergoing increased activity. We therefore tested if there were any functional implications of all or zero AQP4x expression on this neurovascular coupling via wide-field optical intrinsic signal (OIS) imaging of the mouse cortex in anesthetized mice (Rahn et al., 2019). To drive cortical activity, we administered either a subtle (2s) or maximal (6s) left forepaw stimulation and tracked changes to blood flow [total hemoglobin (HbT)] in the contralateral somatosensory cortex. We did not observe any statistical differences in the HbT activation maps (Supplementary Figures 4B, C, H) or in the parameters of the HbT response time course (Supplementary Figures 4D, I, J) to either the 2s or 6s stimulus. We repeated the analysis on oxyhemoglobin (HbO_2_) responses and did not find any statistical differences in response parameters between WT mice, AllX^Hom^, and NoX^Hom^ mice to either 2s or 6s stimulus (Supplementary Figures 4K, L). Thus, within the sensitivity of this assay, neurovascular coupling appears normal across genotypes.

### Cortical vasculature ultrastructural morphology shows subtle differences in AQP4 readthrough mutants

**Figure 5:**
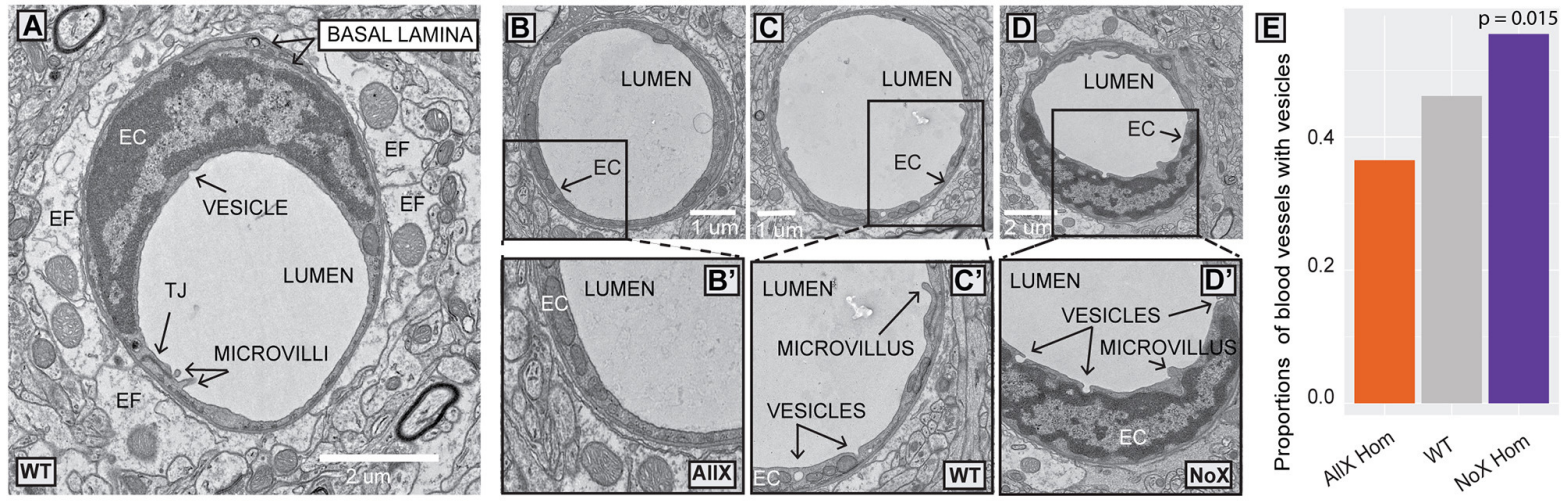
Electron microscopy shows altered proportions of blood vessels with budding endothelial vesicles in AQP4 mutants. **(A)** WT blood vessel ultrastructure: endothelial cell (EC), basal lamina, endfoot (EF), tight junction (TJ), lumen, microvilli, and vesicle. **(B)** AllX blood vessel. **(B')** endothelial cell showing lack of vesicles. **(C)** WT blood vessel. **(C')** endothelial cell showing two vesicles and a microvillus. **(D)** NoX blood vessel. **(D')** endothelial cell with three vesicles and a microvillus. **(E)** Plot depicting significant differences in the number of blood vessels with endothelial vesicles. *N* = 4 mice/genotype and >40 microvessels/mouse. Three-sample test for equality of proportions without continuity correction.

Due to the observed changes in AQP4 localization, we next used electron microscopy to investigate if there were any major changes to cortical gliovascular structures in AllX^Hom^ and NoX^Hom^ mice. We focused our study on capillaries of ≤ 8 μm in diameter to ensure the exclusion of arterioles and venules. All genotypes revealed the expected organization of a lumen surrounded by ECs joined by tight junctions, a thick, electron-dense basal lamina, contacted by a lighter astrocyte endfoot (Figures 5A–D). In the preliminary analysis across genotypes (NoX^Hom^, WT, and AllX^Hom^), we defined a set of features depicted in Supplementary Figure 5 and Supplementary Table 2 for blinded quantification. We found three features that were statistically significant or trending toward significance in the preliminary study: basal lamina “projections without contents,” number of microvilli, and presence of budding endothelial vesicles. These three characteristics were explored in an additional ≥30 blood vessels per mouse (blinded to genotype) with the same criteria as the preliminary study. We observed an *Aqp4x* gene dose-dependent effect on the proportion of ECs containing budding vesicles. NoX^Hom^ mutants had the greatest proportion of blood vessels with endothelial vesicles, followed by WT, with AllX^Hom^ mutants having the least (Figure 5E). Though not explicitly quantified, tight junctions and pericytes were found and appeared normal in all three genotypes (Supplementary Figure 6). Therefore, while the cortical vascular and endfoot structures within AQP4x mutants appear largely normal, some subtle changes were detected. This suggests that there may also be subtle changes to glymphatic clearance and/or BBB functions.

Having detected AQP4x regulation of ECs, we asked if a reciprocal interaction also occurs, i.e., if ECs regulate AQP4x. Brain ECs have been shown to upregulate AQP4 expression and polarization in astrocytes when grown in astrocyte/EC co-cultures (Camassa et al., 2015), and the majority of AQP4 polarized to endfeet *in vivo* is the readthrough version, AQP4x. One possibility suggested by these facts is that ECs induce readthrough of AQP4 in a manner similar to their enhancement of glutamate transporter GLT-1 expression via extracellular signaling to neighboring astrocytes (Martinez-Lozada and Robinson, 2020). Therefore, to determine if ECs promote the upregulation of AQP4x, we grew astrocytes in co-culture or alone under the following culture conditions: astrocytes alone, astrocytes + ECs, astrocytes + neurons, astrocytes + ECs + neurons, and collected protein lysates. We then performed Western blots for AQP4 and AQP4x. Agreeing with previous studies, astrocytes co-cultured with ECs do express AQP4 (Camassa et al., 2015), and ECs alone did not produce AQP4 (Supplementary Figure 7). However, none of the single or co-culture scenarios of astrocytes, ECs, and neurons expressed measurable quantities of AQP4x protein (Supplementary Figure 7). Thus, the presence of ECs alone is not sufficient to induce detectable levels of AQP4 readthrough *in vitro*.

### Blood-brain barrier influx and/or glymphatic efflux are altered in AQP4 NoX^*Hom*^ mice

**Figure 6:**
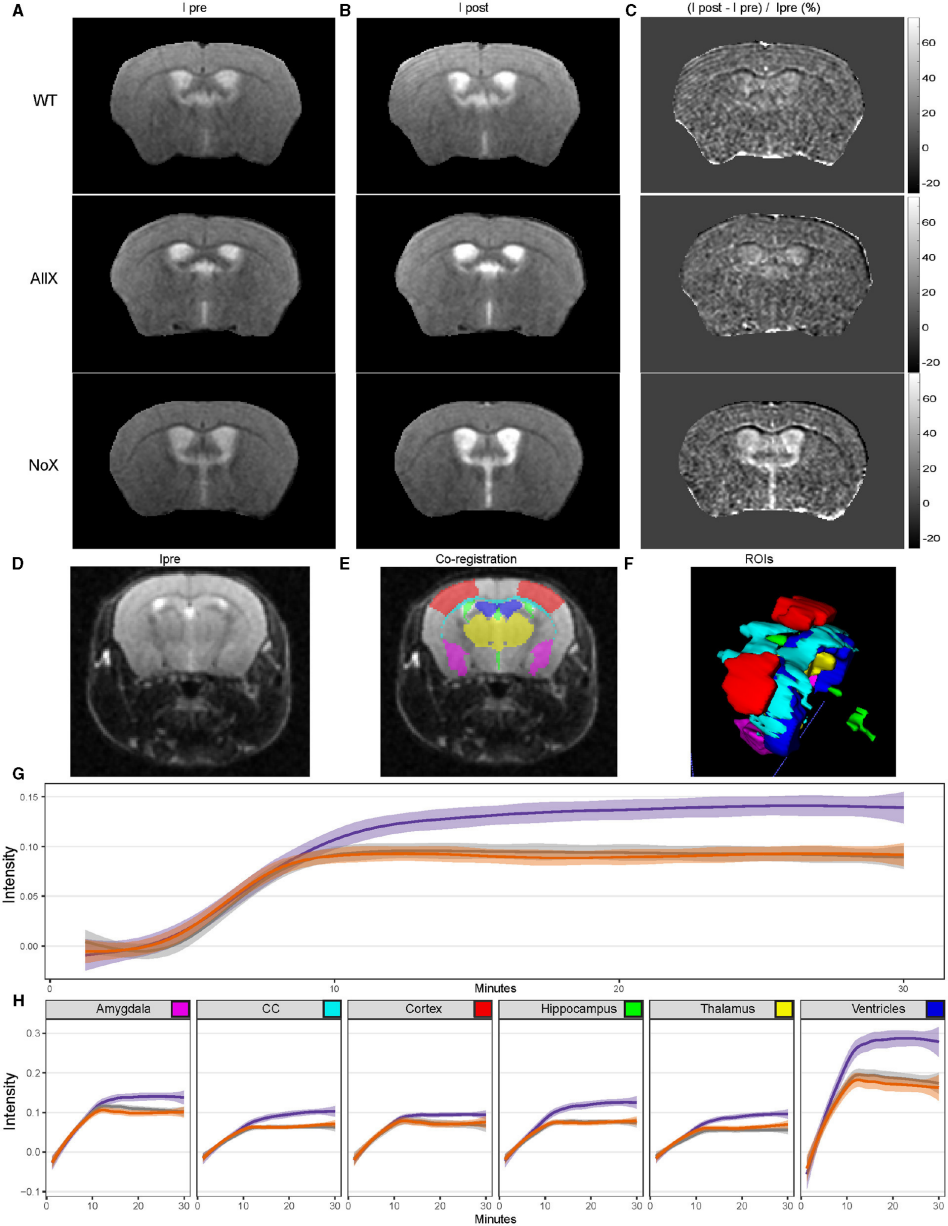
MR measure of the time course of penetration into the brain of systemically administered contrast agents shows NoX^Hom^ mice have altered CNS contrast-agent concentration at steady state. **(A)** Representative images pre-tail-vein injection. **(B)** Post-tail vein injection. **(C)** Normalized change in signal intensity. **(D)** Typical T2-weighted (T2W) transaxial anatomic images. **(D, E)** overlaid with co-registered regions of interest (ROI). **(F)** Three-dimensional rendering of ROIs, including the partial primary somatosensory area (cortex, red), ventricles (blue), hippocampus (green), thalamus (yellow), corpus callosum (CC, cyan), and amygdala (purple). **(G)** GAM curve fit to intensity vs. time for each genotype across combined regions highlights the NoX genotype being more permeable. Shading depicts the 95% confidence interval. The statistically significant effect of genotype by time interaction (Type III Analysis of Variance Table with Satterthwaite's method, *p*-value = 2e-16) was revealed by linear mixed modeling, with mouse and region as nested random effects. The contrast agent (gadolinium) was injected at minute 5. **(H)** GAM curve fit to intensity vs. time for each genotype across individual regions recapitulates the trend seen in **(G)**, with shading again indicating the 95% confidence interval. Color key as in Figure 1.

Finally, we used MRI to assess BBB/“glymphatic” function in *n* = 8–9 animals per genotype by measuring the image-intensity time course following a tail-vein-injected contrast agent. T2-weighted images across a set of preselected regions of interest (ROIs) (Figures 6A–F). Images were acquired once per minute. Exemplary anatomic brain images collected prior to the administration of contrast agent (Ipre) are shown in Figure 6A, left column. After carefully evaluating brain size and structure across genotypes, there are no significant structural differences observed across the three genotypes (NoX^Hom^, WT, and AllX^Hom^). We also examined the proton density across the brain as a measure of overall water content and did not see any differences in water content as a function of genotype (Supplementary Figure 8). ROIs were then auto-segmented, and changes in intensity as a percentage of the pre-contrast image intensity were calculated for six of these: cortex, ventricles, hippocampus, thalamus, corpus callosum, and amygdala. Data were analyzed across all regions (excluding ventricles) in a single omnibus model to examine the influx of contrast over time. We found significantly greater increases in image intensity, reflecting higher levels of contrast agent, specifically in the NoX^Hom^ mice (Figure 6G) at steady state (min 10–30). Examination of individual regions largely follows this pattern (Figure 6H). *Post-hoc* analyses for individual regions are in Supplementary Table 3. Across all brain regions examined, NoX^Hom^ mice showed statistically significant (*p* < 0.05) increases in image intensity post-contrast compared with WT. In comparing NoX^Hom^ vs. AllX^Hom^, some of these ROI intensity increases are statistically significant (NoX^Hom^ > AllX^Hom^) while others trend toward greater increases for NoX mice. These findings reflect either greater BBB permeability (more contrast-agent leakage) or slower glymphatic efflux in the brains of NoX^Hom^ mice compared with either WT or AllX^Hom^.

## Discussion

Here, we generated a novel mouse line that mimics ribosomal readthrough of the stop codon of the *Aqp4* open reading frame to generate greater proportions of the extended variant, AQP4x. We validated this “AllX” line and investigated the AQP4 readthrough's effect on companion proteins and gliovascular structure and function. While AllX and NoX mice had similar body weight, litter distribution, and total brain AQP4 levels to WT animals, levels of AQP4x increased sequentially in a gene-dose-dependent manner (NoX^Hom^ < NoX^Het^ < WT < AllX^Het^ ≤ AllX^Hom^), and though the number of tetrameric forms of AQP4 was equivalent among genotypes, AllX^Hom^ mice did have significantly less OAP formation than WT and NoX mice, indicating that the AQP4x isoform may be less capable of forming these multimeric structures without shorter isoforms present. We also found that higher gene dosages of AQP4x resulted in greater perivascular localization of AQP4, AQP4x, and its binding partner, α-syntrophin; however, perivascular levels of all three of these proteins seemed to mostly saturate beyond a single obligate AQP4 AllX allele, indicating that there may be an upper limit to how much AQP4x can either traffic to or be contained within the endfoot compartment. We then utilized EM to analyze the structure and several features of the endothelium and endfoot compartments but found that the only significant difference between genotypes was in the number of ECs containing budding vesicles. We showed that neurovascular coupling in response to a sensory stimulus was unchanged in the NoX and AllX lines. Finally, we assessed the homozygous lines for BBB integrity and glymphatic function via gadolinium-enhanced MRI and detected an abnormal clearance profile in NoX^Hom^ mice that could be consistent with either an increased influx through the BBB or a deficit in interstitial fluid efflux from the brain.

Several important findings previously illuminated AQP4's role in the regulation of water homeostasis, interstitial fluid efflux, and waste clearance through studies using AQP4 KO mice. AQP4 KO mice show poor outcomes and water movement following vasogenic edema, despite otherwise normal BBB leakiness to macromolecules (Haj-Yasein et al., 2011). Both AQP4 and α-syntrophin KO mice have lower rates of interstitial fluid efflux from the brain (Iliff et al., 2012; Mestre et al., 2018). Our gadolinium-enhanced MRI study suggests that while the AQP4 AllX^Hom^ mice appear equivalent to WTs, the AQP4 NoX^Hom^ mice show a brain-wide phenotype at steady state. As steady state is a function of influx minus efflux, this finding demonstrates that there is either greater permeability or slower clearance of the contrast agent. However, the current study's sampling rate was selected to enable whole-brain analyses and so had an insufficient temporal resolution to fit curves to directly measure the initial influx. Thus, future DCE-MRI studies with higher temporal resolution during the influx and a longer washout period may be able to distinguish these two possibilities. Likewise, analysis of BBB macromolecule permeability using Evan's blue or sodium fluorescein would be a valuable future study to help distinguish between changes to BBB permeability and interstitial fluid efflux. Furthermore, though baseline homeostatic BBB permeability and fluid efflux appear normal in AllX^Hom^ mice, future studies in non-homeostatic conditions such as vasogenic edema or models of Alzheimer's disease may be of interest.

While our data suggest that AQP4x plays a crucial role in localizing AQP4 to the BBB, other studies show that OAP formation could also assist in this role. A recent study using knock-in mice with an OAP-destabilizing A25Q mutation found that OAP depolymerization reduces AQP4 endfoot localization without changing the total AQP4 protein expression (Zhu et al., 2022). KOs of the M23 isoform, which also disrupt OAP formation, had a similar qualitative effect (de Bellis et al., 2021). Perhaps in WT mice, OAPs contribute to the efficient AQP4 localization to the astrocytic endfeet. However, when AQP4x is overexpressed, the cell's physiological need for consistent OAP arrangement might be unnecessary. Another possibility is that the readthrough extended variants assist with endfoot localization, but organization into OAPs stabilizes them at this site. Two hypothesized roles for OAPs are to make AQP4 more immobile and permeable at the blood vessel interface (Furman et al., 2003; de Bellis et al., 2021). Together, our new and previous data show reduced OAP formation, enhanced α-syntrophin recruitment, and amplified clearance of waste when readthrough is promoted (Sapkota et al., 2022), suggesting that AQP4x partners with or interacts with these two roles for OAPs.

The relationship between AQP4, AQP4x, and α-syntrophin is location-dependent, rather than expression-dependent. Previous studies indicate that in α-syntrophin-null mice, AQP4 protein expression is unaffected; however, it becomes more diffuse and its perivascular localization is reduced (Neely et al., 2001; Amiry-Moghaddam et al., 2003). Through immunoprecipitation studies in culture, De Bellis et al. (2017) showed that α-syntrophin-bound AQP4x more readily than non-extended AQP4. However, whether increasing AQP4x levels could alter α-syntrophin endfoot localization was yet unknown. Here, we show that modulation of AQP4 levels also has a significant effect on α-syntrophin endfoot localization, perhaps suggesting that each partner has a stabilizing effect on the other.

We also conducted a careful quantitative evaluation of cortical vascular and astrocyte endfoot structures using electron microscopy in these new lines. We did not observe any differences in vessel diameter or thickness and branching of the basal lamina. While some studies (Zhou et al., 2008) found that AQP4 KO mice have swollen endfeet, we did not find any differences in endfoot size between WT and the AQP4x mutant lines (NoX and AllX). The one change that we did observe was that NoX^Hom^ mice had a greater proportion of ECs containing budding vesicles than WT and AllX^Hom^. This increase in endothelial vesicles could have implications for the integrity of the blood-brain barrier (BBB) as permeability is a function of both the leakiness at tight junctions between ECs and the rate of vesicular transcytosis across the EC (Blanchette and Daneman, 2015). Similar changes have been observed in the dystrophin-deficient mdx mouse, which has more endothelial vesicles and also shows reduced perivascular AQP4 (Nico et al., 2004). Finally, it is important to point out that not much is known about the relationship between endothelial vesicular transcytosis, astrocyte endfeet, and glymphatic clearance, and the occurrence of this phenomenon in two separate models warrants further study.

Polarized AQP4 at the endfoot of astrocytes is a key characteristic of the healthy CNS, as in Alzheimer's disease, AQP4 becomes more depolarized. This reduced localization at the astrocyte endfeet is a hallmark of patients who are susceptible to the disease (Zeppenfeld et al., 2017). For example, researchers found that before full-blown dementia was observed, people who were non-demented but also scored low on the Mini Mental State Examination (MMSE) or high on the Clinical Dementia Rating Scale (CDR) had a reduction in perivascular AQP4 (Simon et al., 2022). This mislocalization is speculated to contribute, thus leading to reduced clearing of toxic debris such as amyloid beta (Xu et al., 2015). We previously showed that across models of neurodegeneration and neuroinflammation, the ratio of AQP4/AQP4x becomes skewed (Sapkota et al., 2019, 2022). Thus, aberrant readthrough rates may play a role in a feedforward loop of disease progression by downregulating clearance as it is most needed. Our results here support the importance of having both AQP4x and AQP4 available for normal brain function as homozygous mice of either allele may have some deficits (e.g., altered influx/efflux in NoX^Hom^ and relative loss of OAPs in AllX^Hom^). Yet, the absence of any detrimental phenotypes in AllX^Het^ mice suggests that moderate upregulation of readthrough may be a safe target for enhancing glymphatic function with the goal of decreasing extracellular accumulation of misfolded protein aggregates. While more directed studies of clearance are still needed, the allelic series generated here should be an important tool going forward for testing these hypotheses. Of course, a caveat to these lines is that the alternation is present throughout development, and thus some compensation across time may have occurred. It would be interesting to engineer new lines that enable an inducible regulation of AQP4x isoform production later in life, mimicking the more abrupt changes seen in injury or disease, to determine the consequences of such acute manipulations.

Finally, there is also an opportunity to study the role of AQP4x in neurodevelopment. It was previously reported that while AQP4 expression can be found in some radial glia during human brain development, AQP4 does not become localized to the blood vessel endfeet in the developing human brain until 25 weeks post-conception (Castañeyra-Ruiz et al., 2022), with similar time courses seen in mice (Fallier-Becker et al., 2014). This, along with Western blot data showing the AQP4x isoform emerges days to weeks after AQP4 expression in mice (Sapkota et al., 2019), indicates that stop codon readthrough of *Aqp4* is a feature of the maturing nervous system. Likewise, AQP4 global null mice have been reported to be vulnerable to induced hydrocephalus (Verkman et al., 2017). The allelic series generated here provides an opportunity to test the role of AQP4x in the timing of vasculogenesis, as the AllX mice will produce the AQP4x isoform precociously, as well as the role of this isoform in hydrocephalus.

## Methods

### Mouse line generation

All procedures involving animals were approved by the Institutional Animal Care and Use Committees at Washington University in St. Louis and the University of Texas at Dallas.

A region distal to the stop codon in the fifth exon of the *Aqp4* gene was targeted with a Cas9 gRNA (5′ ATTGTCTTCCGTATGACTAG 3′). The targeting efficiency of reagents and homologous recombination were confirmed using cell culture. Validated gRNA and Cas9 protein (IDT) were administered into fertilized C57BL6/J oocytes along with single-stranded oligonucleotides carrying homology to the targeted region and providing a TGA-to-TGG, i.e., stop-to-sense, mutation (Seq: 5′ AGCCCGGAGTGGTGCATGTGATTGAC ATTGACCGTGGAGAAGAGAAGAAGGGGAAAGACTCTTCG GGAGAGGTATTGTCTTCCGTATGgCTAGAGGACAGCACTG AAGGCAGAAGAGACTCCCTAGACCTGGCCTCAGATTTCC TGCCACCCATTAAGGAAACAGATTTGTTATAA 3′). After culturing for 1–2 h to ensure viability, the eggs were administered into pseudopregnant surrogate dams for gestation. To screen the resulting pups for the presence of the targeted allele, Illumina sequencing was done on the PCR amplicon amplified with primers flanking the mutation site (5′ GTGATTGACATTGACCGTGGAG 3′ and 5′ GTGTGAAGCAAGAAACCCGCA 3′).

Founders carrying the allele were crossed to WT C57BL/6J mice (JAX Stock No. 000664) to confirm transmission. F1 pups from the lead founder were genotyped by sequencing as above and then bred to generate experimental animals. Subsequent genotyping at each generation was done using the Custom TaqMan SNP Assay (Thermo Fisher Scientific, cat # 4332077). Targeting efficacy was also confirmed with Western blot using AQP4x antibody and pan-AQP4 antibody (Figure 1E).

### Animal husbandry

All mice used in this study were maintained and bred in the vivarium at Washington University in St. Louis on a 12/12-h light/dark cycle with food and water available at all times. Three distinct mouse lines were used on C57BL/6J (WT; RRID:IMSR_JAX:000664) background: AQP4 KO (Thrane et al., 2011), and the custom lines: NoX (Sapkota et al., 2022) and AllX (reported here). Lines were maintained by heterozygous crossings to produce WT, heterozygotes, and homozygotes. Animals were grouped by line and sex at weaning. Tissue was collected from pups for initial genotyping and again after death to verify the genotype as described above (Sapkota et al., 2022).

### Immunofluorescence staining

Age-matched (14–15 weeks old) mice from 6 genotypes (N = 5) were overdosed on isoflurane and then perfused transcardially with 15 ml of PBS, followed by 20 ml of ice-cold 4% paraformaldehyde in PBS. Brains were harvested and transferred to 4% ice-cold paraformaldehyde and rotated end-over-end overnight at 4°C. After overnight fixation, the brains remained at 4°C and were moved through a series of ice-cold sucrose/PBS solutions for cryoprotection as follows: 10% for 4 h, 20% for 4 h, and 30% overnight. The brains were embedded in OCT (Sakura Inc., Torrance, CA, USA), sectioned at 40 μm, and stored at 4°C in PBS+0.01% NaN_3_ until staining.

AQP4/α-syntrophin/CD31: Free-floating sections were washed in 1× phosphate-buffered saline (PBS) three times for 5 min. Slices were incubated in 0.05% Triton-X 100 for 10 min, then blocked in 1× PBS, 5% normal donkey serum, and 0.01% Triton-X 100 for 1 h. Primary antibodies were diluted in a blocking solution, and slices were incubated overnight at 4°C on an orbital shaker. The slices were washed in 1× PBS five times for 5 min each. Secondary antibodies were diluted in a blocking solution (1:1,000), and slices were incubated for 1 h at room temperature, protected from light. The slices were washed three times for 5 min each, incubated in DAPI (1 μg/ml in PBS) for 10 min, and washed for 10 min in 1× PBS. They were then mounted on Super Frost plus slides with ProLong anti-fade reagent.

AQP4/AQP4x/CD31: Staining was performed as above with the following changes: The sections were not washed prior to blocking. The blocking buffer and antibody diluent consisted of PBS with 5% normal donkey serum plus 0.3% Tween-20. A block was performed for 45 min with gentle rotation on an orbital shaker. Three 10-min washes in PBS were performed following overnight primary antibody incubation at 4°C. Secondary antibodies were used at a 1:500 dilution and incubated at room temperature for 1.5 h.

Primary antibodies were 1:1,000 chicken anti-AQP4 (Synaptic Systems 429006), 1:1,000 rabbit anti-AQP4x (made in collaboration with cell signaling PWP2475-1), 1:400 rabbit anti-α-syntrophin (Thermo Fisher Scientific PA5 77702), and 1:50 rat anti-PECAM-1 “CD31” (BD Pharmingen 550274). The anti-AQP4x was a polyclonal antibody generated by immunizing rabbits with a synthetic peptide corresponding to residues surrounding Asp333 of mouse AQPX and purifying the antibody with protein A and peptide affinity chromatography.

Secondary antibodies were Alexa Fluor^®^ 488 AffiniPure Donkey Anti-Chicken IgY (IgG) (H+L) (Jackson Immunoresearch Laboratories, 703-545-155), Donkey anti-Rabbit IgG (H+L) Highly Cross-Adsorbed Secondary Antibody, Alexa Fluor™ 568 (Invitrogen, A10042), and Alexa Fluor^®^ 647 AffiniPure Donkey Anti-Rat IgG (H+L) (Jackson Immunoresearch Laboratories, 712-605-153).

### Imaging and quantification

Thirteen cortical images per mouse were obtained using a Zeiss LSM 700 confocal microscope and Zeiss Zen software. To ensure consistency, all images were taken at a depth of 6 μm below the surface of the section. A custom ImageJ script was written to step through each collected image, assist the user in drawing lines on the various blood vessels visible from the CD31 channel, and automatically export channel values associated with the coordinates along the line to a CSV file. Data associated with this ImageJ macro pipeline are referred to as localization data. One line was drawn per blood vessel in the image, starting from inside the lumen wall of the blood vessel and extending approximately 6–8 μm away from the vessel. Once the data were collected, they were imported into RStudio for analysis. The ImageJ macro code is available on Bitbucket (https://bitbucket.org/jdlabteam/aqp4x_characterization/src/master/).

### Quantified image statistical analysis

The localization data were thresholded to exclude line values exceeding 30 pixels or 4.7 μm. For each channel, a linear mixed model was used to analyze the genotype-by-channel relationship using the lmer command from the R package rstatix. In the following model, *experiment* represents primarily experimenters, and in channels where only one experiment was done (AQP4x and α-syntrophin), this factor is removed: *image* represents the slice, *animal* represents the mouse identification number, *DOP* stands for date of perfusion, *Distance* is the number of pixels away from the start of the vessel, and *Channel* represents either AQP4, CD31, AQP4x, or α-syntrophin.


$$\begin{array}{l} Channel\sim Genotype^{*}Distance+(1|experiment)+(1|animal)\\ +(1|DOP) \end{array}$$

*Post-hoc* analyses on perivascular and parenchymal regions were conducted using the Wilcox test.

### Primary enriched astrocyte cultures

Both sexes of C57BL/6 mouse pups 1–3 days of age were used to prepare enriched primary astrocyte cultures as previously described (Zelenaia et al., 2000). Briefly, mouse brain cortices were dissected, the meninges were removed, and the cortices were dissociated by trypsin and trituration into a single-cell suspension and plated at a density of approximately 2.5 × 10^5^ cells/ml in 75 cm^2^ flasks. Astrocytes were maintained in astrocyte media: Dulbecco's Modified Eagle's Medium (DMEM) (Gibco 11960-014), supplemented with 10% Ham's F12 (Gibco 11765-047), 10% defined heat-inactivated fetal bovine serum (FBS) (HyClone SH30070.03), and 0.24% penicillin/streptomycin (Gibco 15140-122). The media were exchanged every 3–4 days. After 7–10 days (when cell confluency was ~90%), A2B5-positive cells were eliminated using A2B5 hybridoma supernatant (1:50; from the laboratory of Dr. Judy Grinspan, CHOP) and low Tox-M rabbit complement (Cederlane CL3005). After 2–3 days of recovery, the astrocytes were split (1–1.5 surface area to surface area) into 10 cm culture dishes. Under these conditions, >95% of these cells are GFAP-positive.

### Co-cultures

To study astrocyte/endothelial interactions, the mouse brain endothelioma cell line bEND.3 was used (American Type Culture Collection CRL-2299, RRID:CVCL_0170) due to the advantages of a homogeneous population of cells and to reduce the number of animals used. These cells were maintained in endothelial media [DMEM media containing 4.5 g/L D-glucose (Gibco 11960-014) and supplemented with 10% defined heat-inactivated FBS (HyClone, SH30070.3), 4 mM L-glutamine (Gibco 25030-081), and 1 mM sterile filtered sodium pyruvate at 37°C and 5% CO_2_]. This media was exchanged every 3–4 days. To limit genetic drift, cells were never used past passage 30, as per the vendor's recommendation.

In astrocyte/endothelial co-cultures, astrocytes were cultured on top of ECs. From a confluent dish bEND.3 cells were split 1:3 (E). Two days later, when bEND.3 was ~60–70% confluent, astrocytes were replated directly onto empty wells (A) or on top of endothelia (EA).

To study astrocyte/neuronal interactions, rat cortical neurons were obtained from the Neurons R Us service center by the Penn Medicine Translational Neuroscience Center (RRID:SCR_022421). In astrocyte/neuronal co-cultures, neurons were cultured on top of a monolayer of astrocytes. Three-to-four days after the astrocytes were split ~60–70% confluent, neurons were added at a density of 6.67 × 10^5^ neurons/ml on empty poly-D-lysine-coated wells (N), on top of astrocytes (AN), or on top of astrocytes that were co-cultured with ECs (EAN). These cultures were maintained in astrocyte media; one-third of the media was replaced with fresh media every 3–4 days for 10 days.

### Cell lysis and protein quantification

Cells were lysed, and proteins were collected as described previously (Li et al., 2006; Ghosh et al., 2011, 2016). Briefly, the cells were rapidly rinsed in 1× PBS with Ca^2+^/Mg^2+^ and then incubated in ice-cold RIPA on an orbital shaker platform at 4°C for 45 min. The proteins were measured using bicinchoninic acid (Pierce BCA protein assay kit, Thermo Fisher Scientific).

### Western blot

Cell-cultured lysates were diluted in 4× Laemmli sample buffer. A 1:10 dilution of BME to mixture 4× Laemmli sample buffer + sample was made. Notably, 20–40 μg of protein was loaded onto Bio-Rad Mini-Protean TGX gel 7.5% and electrophoresed at 80 V for 10 min and then 120 V for 1 h.

Total brain lysates were homogenized in RIPA buffer and protease inhibitor solution. Samples were then centrifuged at 20,000 × *g* for 20 min at 4°C, and the supernatant was collected. Protein concentration was measured with a BCA assay. A sample of 20 μg was prepared in a 1:1 solution with 2× Laemmli sample buffer. The protein was electrophoresed at 80 V for 10 min and then 120 V for 1 h. Boiling of samples was omitted to prevent AQP4 aggregation for AQP4 assays, while α-syntrophin-probed samples were boiled for 5 min prior to electrophoresis.

Polyvinylidene difluoride (PVDF) membrane was prepared by activating it in 100% methanol for 10 min, washing in DI water for 10 min, and then incubating in transfer buffer (1× SDS-PAGE Running Buffer without SDS, 20% methanol) for 10 min. After electrophoresis, the gel was washed two times in transfer buffer for 10 min to remove salts and SDS. Protein was then transferred to the membrane via a trans-blot SD semi-dry transfer cell (Bio-Rad) and ran according to gel size [(2^*^gel area)/1 h = mA]. For dual fluorescent Western blot, the membrane was dried in a 37°C incubator for 10 min after the transfer was completed, reactivated in 100% methanol for 30 s, and then washed in 1× PBS for 5 min. The membrane was blocked in Li-Cor Intercept^®^ Blocking Buffer-PBS for 1 h. The primary antibody solution consisted of Li-Cor blocking buffer, 1:3,000 guinea pig anti-AQP4 (Synaptic Systems, 429004), and 1:4,000 rabbit anti-AQP4x (Cell Signaling Technologies, 60789) simultaneously, and the membrane was incubated overnight at 4°C. The membrane was washed in 1× PBST four times for 5 min each. The secondary solution consisted of Li-Cor blocking buffer, 1:15,000 Li-Cor IRDye^®^ 680RD donkey anti-guinea pig, and 1:10,000 IRD800 anti-rabbit. Both AQP4 and AQP4x were probed on the same blot. For non-fluorescent probing, the membrane was washed in 1× TBST two times for 5 min each after transfer. A total of 5% milk in 1× TBST was used as a blocking buffer in the 1-h incubation. Primary and secondary dilutions were made in a blocking buffer consisting of 1:2,000 rabbit anti-α-syntrophin (Abcam, ab188873), 1:15,000 mouse anti-GAPDH (Sigma-Aldrich, G8795), 1:2,000 rabbit HRP, and 1:2,000 mouse HRP. The membrane was incubated for 1 h in a secondary solution wrapped in aluminum foil and washed as previously. Images were obtained using MyECL Imager (Thermo Fisher Scientific) or Odyssey M (Li-Cor) and quantified using ImageJ. Equal-sized boxes were constructed around each band, and integrated density values were collected per band.

### Blue native polyacrylamide gel electrophoresis

Brain tissues were lysed in 1× NativePAGE sample buffer (Thermo Fisher Scientific, BN2003). Digitonin and Coomassie Blue G-250 were added to the lystates to the final concentrations of 1 and 0.25%, respectively. Lysates containing 60 μg of total protein were electrophoresed for 3 h at 4°C and 100 V using 3–12% Bis-Tris polyacrylamide gels (ThermoFisher, BN1001BOX) and a running buffer containing 15 mM Bis-Tris, 50 mM Tricine, and 0.002% Coomassie Blue G-250, pH 7. Wet transfer on PVDF membranes was done for 1 h at 4°C and 20 V using a buffer containing 15 mM Bis-Tris and 50 mM Tricine. Membranes were stained with rabbit AQP4 antibody (Cell Signaling, 59678S) and developed for chemiluminescence (Cell Signaling, 6883), as described for the Western blot earlier.

### Transmission electron microscopy

Transmission electron microscopy (TEM) was utilized to investigate quantifiable differences in cortical vascular ultrastructure across three genotypes (AllX, NoX, and WT: N = 4 mice/genotype) of age-matched (11 weeks) and sex-matched mice. EM-grade fixative solution was provided by Washington University's Center for Cellular Imaging (WUCCI). The solution was made by adding 75 ml (Electron Microscopy Sciences) of 16% paraformaldehyde and 30 ml of 50% glutaraldehyde to 300 ml of a WUCCI-provided 2× cacodylate buffer. In all, 195 ml of water was added to bring the total fixative volume to 600 ml. The fixative solution was mixed thoroughly and warmed to 37°C immediately preceding perfusion. The final concentrations for the mixed fixative were as follows: 2.5% glutaraldehyde, 2% paraformaldehyde, and 0.15 M cacodylate buffer pH 7.4 with 2 mM CaCl_2_. Mice were anesthetized by a 2-min exposure to isoflurane and transcardially perfused for 2 min using a Dynamax peristaltic pump with a pump speed of 31 (~6 ml/min) with filtered 37°C Krebs-Ringer buffer (NaCl 120 mM, KCl 6 mM, NaHCO_3_ 15.5 mM, MgCl_2_ 1.2 mM, NaH_2_PO_4_ 1.2 mM, dextrose 11.2 mM, CaCl_2_ 2.5 mM) containing heparin (20 units/ml) followed by 6 min (~20 ml) perfusion of 37°C fixative solution. Bodies became very rigid and were decapitated. The brains were carefully extracted and transferred to 14 ml Einstein tubes containing 5–10 ml RT fixative solution and transferred to 4°C for overnight fixation with gentle rotation. The next day, samples were taken to WUCCI for sample preparation for EM. The preparation was performed by WUCCI staff, and their methods were provided as follows: post-fixation, samples of the mouse brain were cut into 100-μm-thick sections using a vibratome (Leica VT1200S, Vienna, Austria). Sections containing relevant ROIs were rinsed in 0.15 M cacodylate buffer containing 2 mM calcium chloride three times for 10 min each, followed by a secondary fixation in 1% osmium tetroxide and 1.5% potassium ferrocyanide in 0.15 M cacodylate buffer containing 2 mM calcium chloride for 1 h in the dark. The samples were then rinsed three times in ultrapure water for 10 min each and *en bloc* stained with 2% aqueous uranyl acetate overnight at 4°C in the dark. After another four washes in ultrapure water, the samples were dehydrated in a graded ethanol series (30, 50, 70, 90, 100% x3) for 10 min each step. Once dehydrated, samples were infiltrated with LX112 resin (Electron Microscopy Sciences, Hatfield, PA), flat embedded, and polymerized at 60°C for 48 h. Post-curing, specific ROIs were excised and mounted on a blank epoxy stub for sectioning. In all, 70 nm sections were then cut, post-stained with 2% aqueous uranyl acetate and Sato's lead, and imaged on a TEM (JEOL JEM-1400 Plus) at an operating voltage of 120 kV. Two grids per mouse across three genotypes (NoX^Hom^, WT, and AllX^Hom^) were used to obtain, at minimum, 10 images of cortical blood vessels and the surrounding perivascular astrocytic processes (PVAP's = endfeet) plus additional high magnification images of each blood vessel wall and perivascular structures, resulting in over 800 images for our initial investigation. We focused our image acquisition on blood vessels that were observed to be cut in the transverse plane, rendering them more circular in shape; beyond that, cortical blood vessels were chosen at random, regardless of size (or type of vessel). The following ultrastructure morphological characteristics were chosen for initial quantification blinded to genotype: vessel diameter (shortest/longest), widest endfoot (tangential/radial), thick basal lamina (Y/N), number of basal lamina “projections with contents,” number of basal lamina “projections without contents,” branching in lamina (Y/N), and presence of budding vesicles in endothelium (Y/N). Observations were statistically analyzed with all data normalized to blood vessel circumference to rule out differences in observations due to blood vessel type (Figure 5, Supplementary Figures 4, 5, Supplementary Table 2). After analysis of the initial study, the same samples were used to make new grids. In our subsequent investigation, we imaged ~30 blood vessels/mouse using the same criteria as the pilot study. However, upon acquisition, we focused quantification on the following: basal lamina projections, microvilli, and budding endothelial vesicles, as these were the characteristics that were statistically significant or trending toward significance in the initial EM quantification. Quantification was again blinded to mouse genotype with data normalized to blood vessel circumference.

### OIS imaging

Mice were fitted with transparent chronic optical windows made of plexiglass as previously described (Rahn et al., 2019). Briefly, each mouse received <7 mg/kg 0.5% lidocaine and 0.01 mg/kg Buprenorphine SR via subcutaneous injection prior to surgery. The mice were then anesthetized using isoflurane and maintained at 37°C using a heating pad; their heads were shaved. To adhere the plexiglass window to the dorsal cranium, an incision was made along the midline of the scalp to retract the skin and expose an approximately 1.1 cm^2^ dorsal cortical field of view. The plexiglass window was adhered to the dorsal cranium using Metabond clear dental cement (C&B-Metabond, Parkell Inc., Edgewood, NY). The mice were returned to their cages to recover for at least 72 h before data acquisition was performed.

In accordance with our previously published anesthetized OIS acquisition protocol (Wright et al., 2017; Rahn et al., 2019), anesthesia was induced via an intraperitoneal injection of a ketamine/xylazine cocktail (86.9 mg/kg ketamine and 13.4 mg/kg xylazine). The mouse was inserted into the imaging apparatus and maintained at 37°C using a heating pad. Sequential illuminations by four LEDs (470, 530, 590, and 625 nm) allowed for measuring hemodynamic fluctuations (Wright et al., 2017). An sCMOS camera (Zyla 5.5, Andor Technologies; Belfast, Northern Ireland, United Kingdom) was synchronized with the LED illuminations and captured frames at a rate of 16.8 Hz per LED channel.

### Electrical forepaw stimulation

Electrical stimulation was generated by an isolated pulse stimulation (Model 2100, A-M Systems) and administered to the left forepaw via microvascular clips (Roboz Surgical Instrument Co.). To evaluate two different forms of hemodynamic response to somatosensory stimuli, we used a block design consisting of both 2s and 6s stimuli in 4.5-min runs. Each 4.5-min run consisted of six 45s blocks in which there was an initial 5s baseline, followed by alternating 2s or 6s stimulation (frequency: 3 Hz, pulse duration: 300 μs, current: 0.75 mA), and 38s or 34s rest. The blocks containing 2s or 6s stimulations within each run were block averaged separately for analysis.

### OIS data processing and analysis

Optical imaging data were processed using MATLAB following an analysis pipeline described elsewhere (Wright et al., 2017) with modifications. Briefly, a binary brain mask was created for each mouse by manually tracing the mouse dorsal cortex within the field of view. Background light levels were subtracted, and each pixel's individual time trace was temporally and spatially detrended. Using the changes in reflectance in the 530, 590, and 625 nm LED channels, a modified Beer–Lambert Law was solved to generate fluctuations in oxy- and deoxyhemoglobin concentration, as described previously (White et al., 2011). Changes in total hemoglobin concentrations were the sum of changes in oxy- and deoxyhemoglobin concentrations. Global signal regression was performed by regressing the average of all time traces of pixels within the mask from each pixel's time trace. Individual runs were excluded from analysis if raw LED light levels showed >1% variance across the run or if visual inspections showed a clear lack of activation in the somatosensory cortex. Stimulation data were filtered over the 0.009–0.25 Hz frequency band.

### MRI assessment of contrast agent accumulation in the brain

MRI experiments were performed using a 4.7-T small-animal MR scanner (Agilent/Varian, Santa Clara, CA). Data were collected with a laboratory-built, actively-decoupled transmit/receive coil pair: 7.5-cm ID volume transmitter coil and 1.6-cm (OD) surface receiver coil. Mice were placed in a prone position in a customized cradle and anesthetized with isoflurane (1.2%/O_2_). Mouse body temperature was controlled at 37.0 ± 0.5°C with a physiologic monitoring unit (Small Animal Instruments, Stony Brook, NY) employing the combination of a warm water pad and warm air blown through the bore of the magnet.

T2-weighted (T2W) transaxial anatomic images were collected before and after dynamic contrast-enhanced (DCE) MRI experiments (pre-contrast, Ipre; post-contrast, Ipost) with a 2D fast-spin-echo (fsems) sequence: matrix size, 128 × 128; FOV, 16 × 16 mm^2^; slice thickness, 0.5 mm; resolution, 0.125 × 0.125 × 0.5 mm^3^; 21 slices; echo train length, 4; Kzero, 4; TR, 1.5 s; effective TE, 52 ms; 4 averages. T1-weighted transaxial DCE images were collected with a 2D gradient-echo (gems) sequence: matrix size, 64 × 64; FOV, 16 × 16 mm^2^; slice thickness, 0.5 mm; resolution, 0.25 × 0.25 × 0.5 mm^3^; 21 slices; TR, 150 ms; TE, 2.3 ms; flip angle, 60°; 8 averages per time frame (1.25 min), with a total of 24 time frames (30 min) defining the full DCE experiment. Dotarem^®^, at a dose of 1 mmole/g body weight, was injected over 5 s through a tail-vein catheter following the fifth time frame (Ge et al., 2019, 2022). We note the absence of a washout phase during the experiment in any of the genotypes or brain regions over the 30-min time course of the experiment.

### MR image processing and segmentation

Anatomic MRI images (I_pre_ and I_post_) were read into MATLAB, smoothed with a Gaussian filter (Sigma 0.75), and converted into NIfTI format (.nii). The I_pre_ (aka T2W) images were skull-stripped manually and registered to a published mouse brain atlas (Dorr et al., 2008) using the Advanced Neuroimaging Tools (ANTs) (Avants et al., 2014). ROIs were auto-segmented with ANTs based on the label map associated with the mouse brain atlas. Typical ROIs were combined in MATLAB as a NIfTI file. Brain ROIs considered in this work include the partial primary somatosensory area (GM, cortex), ventricles, hippocampus, thalamus, corpus callosum (CC), and amygdala (Figure 5). The ratio I_ratio_ = 100(%) ^*^ (I_post_ – I_pre_)/I_pre_ was computed in MATLAB. DCE data were zero-padded to 128 × 128 and smoothed with a Gaussian filter (Sigma 0.75), and the normalized DCE signal at each time point, S_j_' (j' = 1, 20), was calculated as S_j_' = (S_j_ – S_0_)/S_0_, in which S_0_ is the average of the first four-time frames before tail-vein injection of Dotarem^®^. DCE data (S_j_') across each ROI were extracted for subsequent statistical analysis using MATLAB. All MRI images and DCE data ROIs were inspected and confirmed with ITK-SNAP (Yushkevich et al., 2006). Anatomic images collected before and after the DCE experiment were compared to ensure that the mouse did not move during the imaging pipeline. Two of the 28 datasets collected were discarded due to motion.

## Data availability statement

The original contributions presented in the study are included in the article/Supplementary material, further inquiries can be directed to the corresponding authors, or found at this link: https://bitbucket.org/jdlabteam/aqp4x_characterization/src/master/.

## Ethics statement

The animal study was approved by Institutional Animal Care and Use Committees at Washington University in St. Louis and the University of Texas at Dallas. The study was conducted in accordance with the local legislation and institutional requirements.

## Author contributions

DS: Conceptualization, Formal analysis, Funding acquisition, Investigation, Methodology, Supervision, Writing – original draft, Writing – review & editing. SM: Formal analysis, Investigation, Methodology, Writing – original draft. KM: Formal analysis, Investigation, Methodology, Writing – original draft. SF: Formal analysis, Writing – original draft. SC: Formal analysis, Investigation, Methodology, Writing – original draft. ZS: Formal analysis, Investigation, Methodology, Writing – original draft. XG: Formal analysis, Investigation, Methodology, Writing – original draft. JE: Investigation, Methodology, Writing – original draft. SG: Investigation, Methodology, Writing – original draft. AB: Investigation, Methodology, Writing – original draft. MV: Investigation, Writing – original draft, Formal analysis, Writing – review & editing. JG: Formal analysis, Supervision, Writing – original draft. JC: Formal analysis, Supervision, Writing – original draft. ZM-L: Formal analysis, Investigation, Writing – original draft. MC-S: Formal analysis, Writing – original draft. JD: Conceptualization, Formal analysis, Funding acquisition, Supervision, Writing – original draft, Writing – review & editing.
