# Supplementary material for: Evaluation of gliovascular functions of AQP4 readthrough isoforms
Source: Front Cell Neurosci. 2023 Nov 23;17:1272391. doi: 10.3389/fncel.2023.1272391 (PMC10701521; doi:10.3389/fncel.2023.1272391)
Supplement: Supplementary file 1 [file Table_1.pdf]

SUPPLEMENTARY MATERIAL

Evaluation of gliovascular functions of Aqp4 readthrough isoforms

Shayna M. Mueller*[1,2], Kelli McFarland White*[1,2], Stuart B. Fass[1,2], Siyu Chen[1,2,3], Zhan Shi[4], Xia Ge[3,6], John A. Engelbach[3,6], Seana H Gaines[3], Annie R Bice[3], Michael J. Vasek[1,2], Joel R. Garbow[3,6], Joseph P. Culver[3,7,8,9,10], Zila Martinez-Lozada[11], Martine Cohen-Salmon[12], Joseph D. Dougherty[++1,2,6], Darshan Sapkota[++4,5]

Supplemental Table 1: IF statistical summary

	AllX Het (n = 2236, Mice = 5)	AllX Hom (n = 2177, Mice = 5)	KO (n = 1174, Mice = 5)	NoX Het (n = 1220, Mice = 5)	NoX Hom (n = 1274, Mice = 5)	WT (n = 2018, Mice = 5)	Total (n = 10099, Mice = 30)
**CD31**							
Mean (SD)	7560 (4730)	8490 (4960)	9900 (6410)	11000 (5800)	11500 (6510)	10900 (6210)	9600 (5860)
Median [Min, Max]	6450 [645, 32900]	7430 [827, 30100]	8210 [1100, 41400]	9970 [1370, 42600]	10700 [1090, 35200]	9680 [772, 42300]	8380 [645, 42600]
**AQP4**							
Mean (SD)	8810 (8380)	9660 (9400)	2280 (2710)	5060 (4790)	3140 (3440)	10100 (8950)	6950 (7810)
Median [Min, Max]	6170 [304, 54500]	6970 [78.9, 56900]	1270 [136, 18700]	3500 [358, 41400]	1600 [275, 27500]	7370 [349, 47900]	3940 [78.9, 56900]
Missing	465 (20.8%)	546 (25.1%)	0 (0%)	0 (0%)	0 (0%)	465 (23.0%)	1476 (14.6%)
**AQP4x**							
Mean (SD)	22100 (12900)	20800 (11800)	928 (445)	6440 (4620)	992 (357)	17300 (10100)	14000 (12700)
Median [Min, Max]	21900 [616, 61500]	20400 [600, 59700]	877 [223, 5760]	5840 [354, 25700]	964 [170, 5620]	16000 [90.3, 54600]	11400 [90.3, 61500]
Missing	1121 (50.1%)	1083 (49.7%)	624 (53.2%)	563 (46.1%)	637 (50.0%)	838 (41.5%)	4866 (48.2%)
**α-syn**							
Mean (SD)	8000 (4850)	6700 (4580)	2740 (1750)	3630 (1990)	2670 (1270)	3960 (2410)	4730 (3850)
Median [Min, Max]	6930 [710, 32700]	5530 [670, 31800]	2260 [487, 9530]	3180 [538, 13200]	2460 [506, 11200]	3520 [671, 16200]	3520 [487, 32700]
Missing	1520 (68.0%)	1596 (73.3%)	550 (46.8%)	657 (53.9%)	637 (50.0%)	1591 (78.8%)	6551 (64.9%)
