# Supplementary material for: Evaluation of gliovascular functions of AQP4 readthrough isoforms
Source: Front Cell Neurosci. 2023 Nov 23;17:1272391. doi: 10.3389/fncel.2023.1272391 (PMC10701521; doi:10.3389/fncel.2023.1272391)
Supplement: Supplementary file 2 [file Table_2.pdf]

SUPPLEMENTARY MATERIAL

Evaluation of gliovascular functions of Aqp4 readthrough isoforms

Shayna M. Mueller*[1,2], Kelli McFarland White*[1,2], Stuart B. Fass[1,2], Siyu Chen[1,2,3], Zhan Shi[4], Xia Ge[3,6], John A. Engelbach[3,6], Seana H Gaines[3], Annie R Bice[3], Michael J. Vasek[1,2], Joel R. Garbow[3,6], Joseph P. Culver[3,7,8,9,10], Zila Martinez-Lozada[11], Martine Cohen-Salmon[12], Joseph D. Dougherty[++1,2,6], Darshan Sapkota[++4,5]

Supplemental Table 2: Statistical summary, n, and mean differences for all electron microscopy analyses.

Preliminary

	WT (N=35)	NoX Hom (N=41)	AllX Hom (N=41)	Total (N=117)	Model / Statistical Test	P value
**Basal Lamina Projections with Contents (#) (presumptive pericytes)**					genotype + (1|mouse_ID/Grid)	0.751
Mean (SD)	0.229 (0.877)	0.220 (0.525)	0.0976 (0.436)	0.179 (0.624)		
Median [Min, Max]	0 [0, 5.00]	0 [0, 2.00]	0 [0, 2.00]	0 [0, 5.00]		
**Widest endfoot radial (um)**					genotype + (1|mouse_ID/Grid)	0.5282
Mean (SD)	2.13 (1.67)	2.21 (1.84)	1.60 (1.22)	1.97 (1.60)		
Median [Min, Max]	1.60 [0.336, 7.95]	1.59 [0.211, 7.79]	1.29 [0.121, 6.38]	1.54 [0.121, 7.95]		
**Widest endfoot tangential (um)**					genotype + (1|mouse_ID/Grid)	0.5282
Mean (SD)	6.63 (3.93)	5.42 (2.74)	4.78 (1.93)	5.55 (3.00)		
Median [Min, Max]	5.14 [2.90, 18.4]	4.99 [1.05, 12.8]	4.46 [0.526, 9.97]	4.79 [0.526, 18.4]		
**Basal Lamina projections without Contents (#)**					genotype + (1|mouse_ID/Grid)	0.1831
Mean (SD)	0.771 (2.41)	0.488 (1.45)	0.0488 (0.218)	0.419 (1.59)		
Median [Min, Max]	0 [0, 10.0]	0 [0, 6.00]	0 [0, 1.00]	0 [0, 10.0]		
**Thick basal lamina (Yes / No)**					genotype + (1|mouse_ID)	0.953
No	15 (42.9%)	19 (46.3%)	19 (46.3%)	53 (45.3%)		
Yes	20 (57.1%)	22 (53.7%)	22 (53.7%)	64 (54.7%)		

**lamina branching (Yes / No)**					genotype + (1|mouse_ID)	0.2132
No	1 (2.9%)	7 (17.1%)	5 (12.2%)	13 (11.1%)		
Yes	34 (97.1%)	34 (82.9%)	36 (87.8%)	104 (88.9%)		
**Microvilli (#)**					genotype + (1|mouse_ID/Grid)	0.1396
Mean (SD)	5.11 (6.21)	4.71 (6.63)	2.32 (2.23)	3.99 (5.45)		
Median [Min, Max]	2.00 [0, 24.0]	3.00 [0, 38.0]	2.00 [0, 9.00]	2.00 [0, 38.0]		
**Budding Endothelial Vesicles (Yes / No)**					genotype + (1|mouse_ID)	0.2915
No	12 (34.3%)	15 (36.6%)	9 (22.0%)	36 (30.8%)		
Yes	23 (65.7%)	26 (63.4%)	32 (78.0%)	81 (69.2%)		

Replication

	WT (Mice = 4, Vessels =117)	NoX Hom (Mice = 4, Vessels=119)	AllX Hom (Mice = 4, Vessels=115)	Total (Mice = 12, Vessels= 351)	Model / Statistical Test	P value
**Microvilli (#)**					genotype + (1|mouse_ID)	0.1536
Mean (SD)	2.47 (3.85)	1.82 (2.48)	1.57 (2.09)	1.96 (2.93)		
Median [Min, Max]	1.00 [0, 20.0]	1.00 [0, 19.0]	1.00 [0, 13.0]	1.00 [0, 20.0]		
**Number of Basal Lamina projections without Contents**					genotype + (1|mouse_ID)	0.1886
Mean (SD)	0.111 (0.728)	0.00840 (0.0917)	0.0435 (0.244)	0.0541 (0.447)		
Median [Min, Max]	0 [0, 7.00]	0 [0, 1.00]	0 [0, 2.00]	0 [0, 7.00]		
**Budding Endothelial Vesicles**					3-sample test for equality of proportions without continuity correction	0.01468
No	63 (53.8%)	53 (44.5%)	73 (63.5%)	189 (53.8%)		
Yes	54 (46.2%)	66 (55.5%)	42 (36.5%)	162 (46.2%)		
