# Supplementary material for: Evaluation of gliovascular functions of AQP4 readthrough isoforms
Source: Front Cell Neurosci. 2023 Nov 23;17:1272391. doi: 10.3389/fncel.2023.1272391 (PMC10701521; doi:10.3389/fncel.2023.1272391)
Supplement: Supplementary file 3 [file Table_3.pdf]

SUPPLEMENTARY MATERIAL

Evaluation of gliovascular functions of Aqp4 readthrough isoforms

Shayna M. Mueller*[1,2], Kelli McFarland White*[1,2], Stuart B. Fass[1,2], Siyu Chen[1,2,3], Zhan Shi[4], Xia Ge[3,6], John A. Engelbach[3,6], Seana H Gaines[3], Annie R Bice[3], Michael J. Vasek[1,2], Joel R. Garbow[3,6], Joseph P. Culver[3,7,8,9,10], Zila Martinez-Lozada[11], Martine Cohen-Salmon[12], Joseph D. Dougherty[++1,2,6], Darshan Sapkota[++4,5]

Supplemental Table 3: MRI statistical summaries

Genotype	Cortex	Ventricles	Hippocampus	Thalamus	Corpus Collosom	Amygdala
			**Mean % Change (s.d.):**			
Wildtype (n=9)	11.9 (5.4)	18.0 (10.3)	10.0 (4.4)	10.9 (4.7)	13.2 (4.5)	12.3 (3.8)
NoX (n=9)	18.0 (4.4)	31.7 (11.7)	18.2 (4.2)	18.2 (3.7)	19.8 (5.1)	17.2 (3.7)
AllX (n=8)	16.4 (2.9)	18.6 (5.8)	13.8 (1.9)	15.2 (1.6)	16.3 (2.0)	14.4 (1.7)
			**P value**			
NoX vs. WT	0.025	0.025	0.002	0.004	0.015	0.018
NoX vs. AllX	0.417	0.016	0.023	0.061	0.108	0.072
WT vs. AllX	0.061	0.895	0.045	0.036	0.103	0.191

Post hoc analyses of MRI measures of differential contrast enhancement (DCE) by region. DCE data collected over 24 timeframes of 1.25 min. The greater increase in image intensity following administration of Gd-based contrast agent for NoX vs. AllX or WT mice is highlighted by calculating the difference in intensities prior to injection (I_{Pre}) and after injection (I_{Post}) and correcting for baseline differences between regions (divide by I_{Pre}). i.e., **(I_{Post} - I_{Pre}) / (I_{Pre}) *100.** There is higher contrast agent in NoX mice in all these regions at minutes 10-30.
