# Supplementary material for: Evaluation of gliovascular functions of AQP4 readthrough isoforms
Source: Front Cell Neurosci. 2023 Nov 23;17:1272391. doi: 10.3389/fncel.2023.1272391 (PMC10701521; doi:10.3389/fncel.2023.1272391)
Supplement: Supplementary file 4 [file Image_1.pdf]

SUPPLEMENTARY MATERIAL

Evaluation of gliovascular functions of Aqp4 readthrough isoforms

Shayna M. Mueller*[1,2], Kelli McFarland White*[1,2], Stuart B. Fass[1,2], Siyu Chen[1,2,3], Zhan Shi[4], Xia Ge[3,6], John A. Engelbach[3,6], Seana H Gaines[3], Annie R Bice[3], Michael J. Vasek[1,2], Joel R. Garbow[3,6], Joseph P. Culver[3,7,8,9,10], Zila Martinez-Lozada[11], Martine Cohen-Salmon[12], Joseph D. Dougherty[++1,2,6], Darshan Sapkota[++4,5]

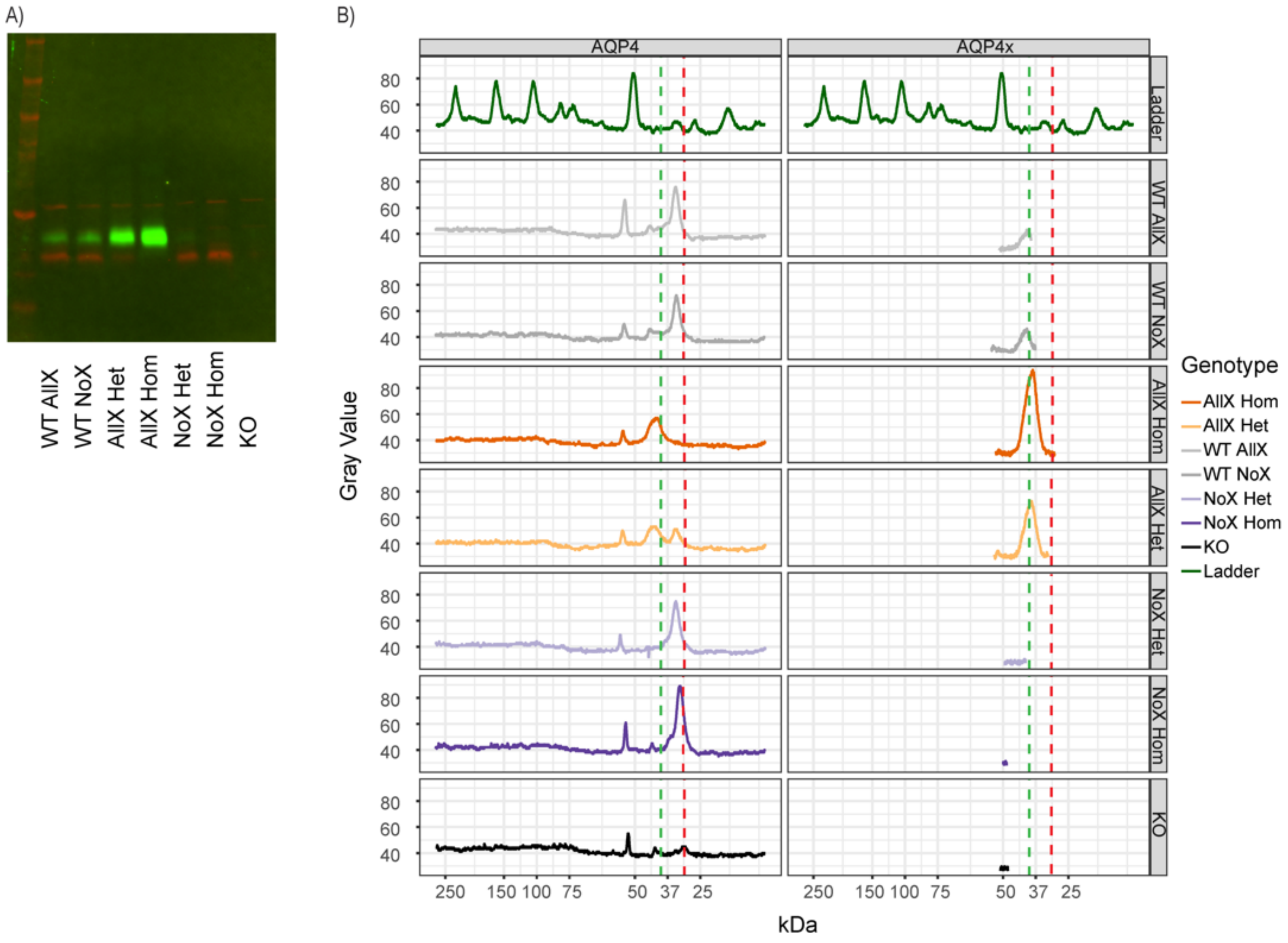

Supplemental Figure 1: AQP4x protein expression is altered based on genotype. A) AQP4 (red) and AQP4x (green) simultaneously probed in western blot. Increased AQP4x protein expression is observed in AllX variants, while a decrease is seen in NoX variants. **B)** A linear peak plot of the fluorescent western was derived by drawing a line down each lane and plotting signal intensity by position. Ladder shown at top for reference. Left panel is AQP4, right panel is AQP4x. Dotted lines show expected sizes of the two isoforms AQP4 (red) and AQP4x (green).
