# Supplementary material for: Evaluation of gliovascular functions of AQP4 readthrough isoforms
Source: Front Cell Neurosci. 2023 Nov 23;17:1272391. doi: 10.3389/fncel.2023.1272391 (PMC10701521; doi:10.3389/fncel.2023.1272391)
Supplement: Supplementary file 5 [file Image_2.pdf]

SUPPLEMENTARY MATERIAL

Evaluation of gliovascular functions of Aqp4 readthrough isoforms

Shayna M. Mueller*[1,2], Kelli McFarland White*[1,2], Stuart B. Fass[1,2], Siyu Chen[1,2,3], Zhan Shi[4], Xia Ge[3,6], John A. Engelbach[3,6], Seana H Gaines[3], Annie R Bice[3], Michael J. Vasek[1,2], Joel R. Garbow[3,6], Joseph P. Culver[3,7,8,9,10], Zila Martinez-Lozada[11], Martine Cohen-Salmon[12], Joseph D. Dougherty[++1,2,6], Darshan Sapkota[++4,5]

A)

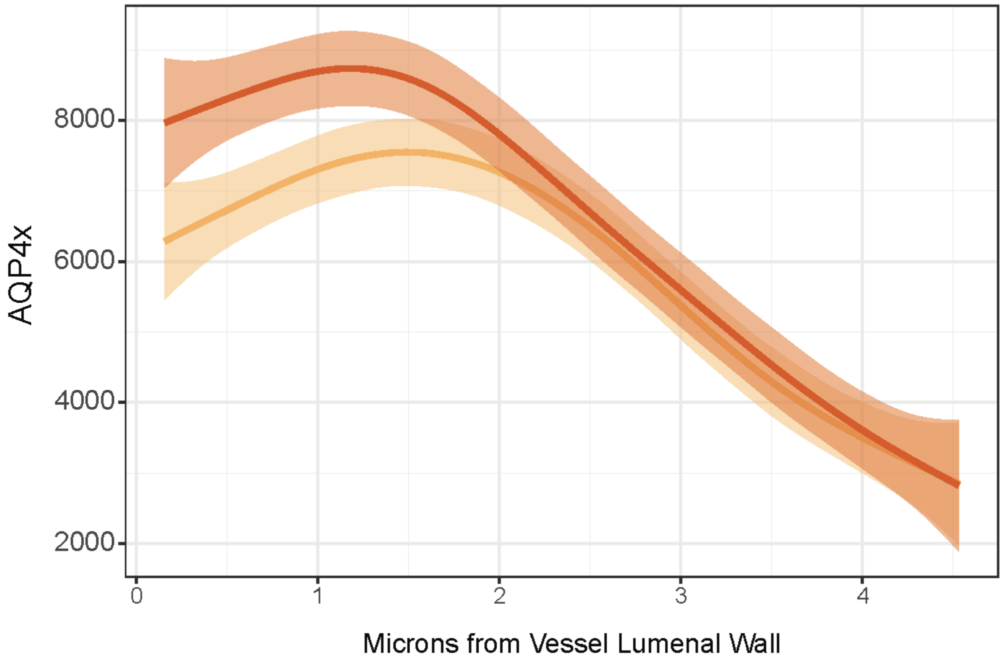

AQP4x
8000
6000
4000
2000
0
1
2
3
4
Microns from Vessel Lumenal Wall

B)

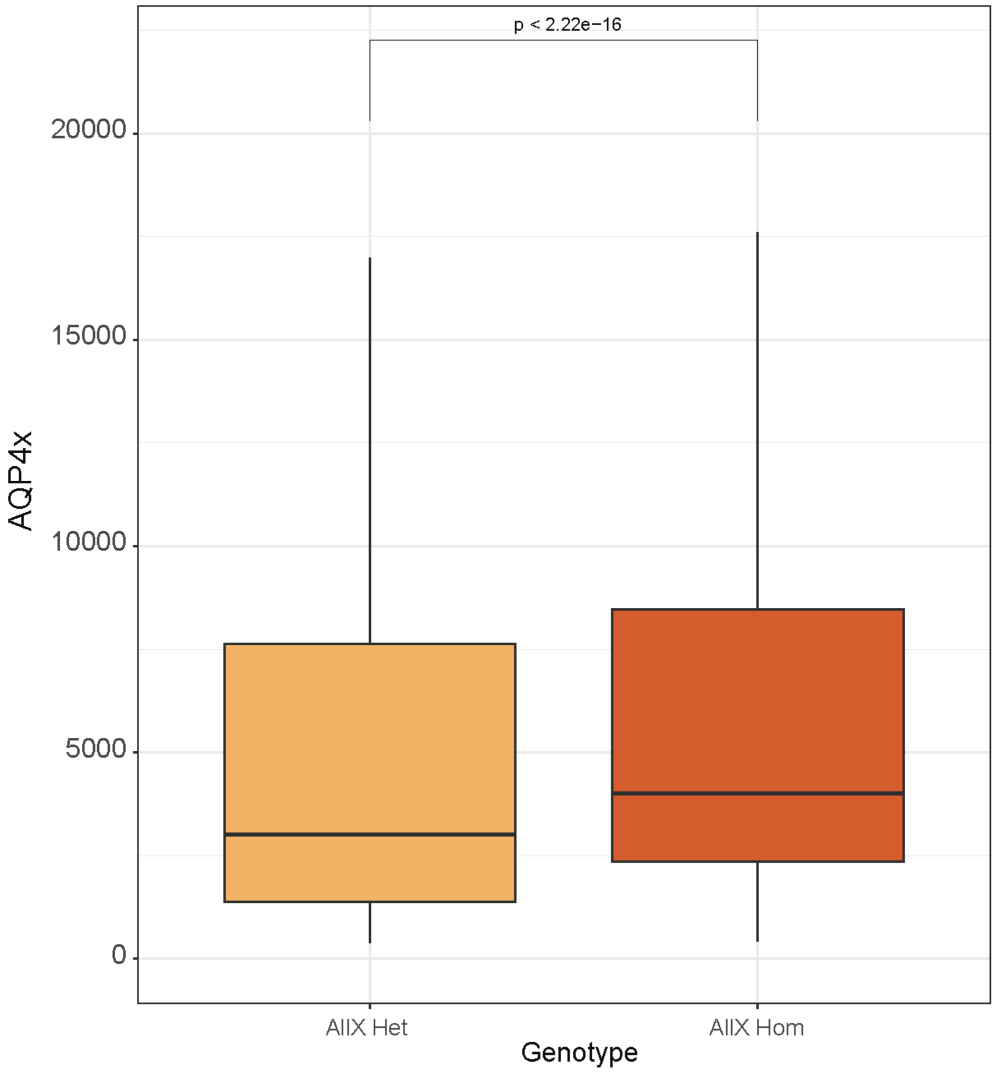

p < 2.22e−16
AQP4x
20000
15000
10000
5000
0
AIIX Het
AIIX Hom
Genotype

Supplemental Figure 2: Reimaging with no AQP4x saturation indicates modest but significant differences between AIIX^{Hom} and AIIX^{Het} A) Loess curve fit to distribution of AQP4x fluorescent signal. **B)** Boxplot with outliers not shown depicts perivascular AQP4x levels are slightly higher in AIIX Hom genotype, significant p value < 2.22e-16 by wilcox test.
