# Supplementary material for: Evaluation of gliovascular functions of AQP4 readthrough isoforms
Source: Front Cell Neurosci. 2023 Nov 23;17:1272391. doi: 10.3389/fncel.2023.1272391 (PMC10701521; doi:10.3389/fncel.2023.1272391)
Supplement: Supplementary file 7 [file Image_4.pdf]

SUPPLEMENTARY MATERIAL

Evaluation of gliovascular functions of Aqp4 readthrough isoforms

Shayna M. Mueller*[1,2], Kelli McFarland White*[1,2], Stuart B. Fass[1,2], Siyu Chen[1,2,3], Zhan Shi[4], Xia Ge[3,6], John A. Engelbach[3,6], Seana H Gaines[3], Annie R Bice[3], Michael J. Vasek[1,2], Joel R. Garbow[3,6], Joseph P. Culver[3,7,8,9,10], Zila Martinez-Lozada[11], Martine Cohen-Salmon[12], Joseph D. Dougherty[++1,2,6], Darshan Sapkota[++4,5]

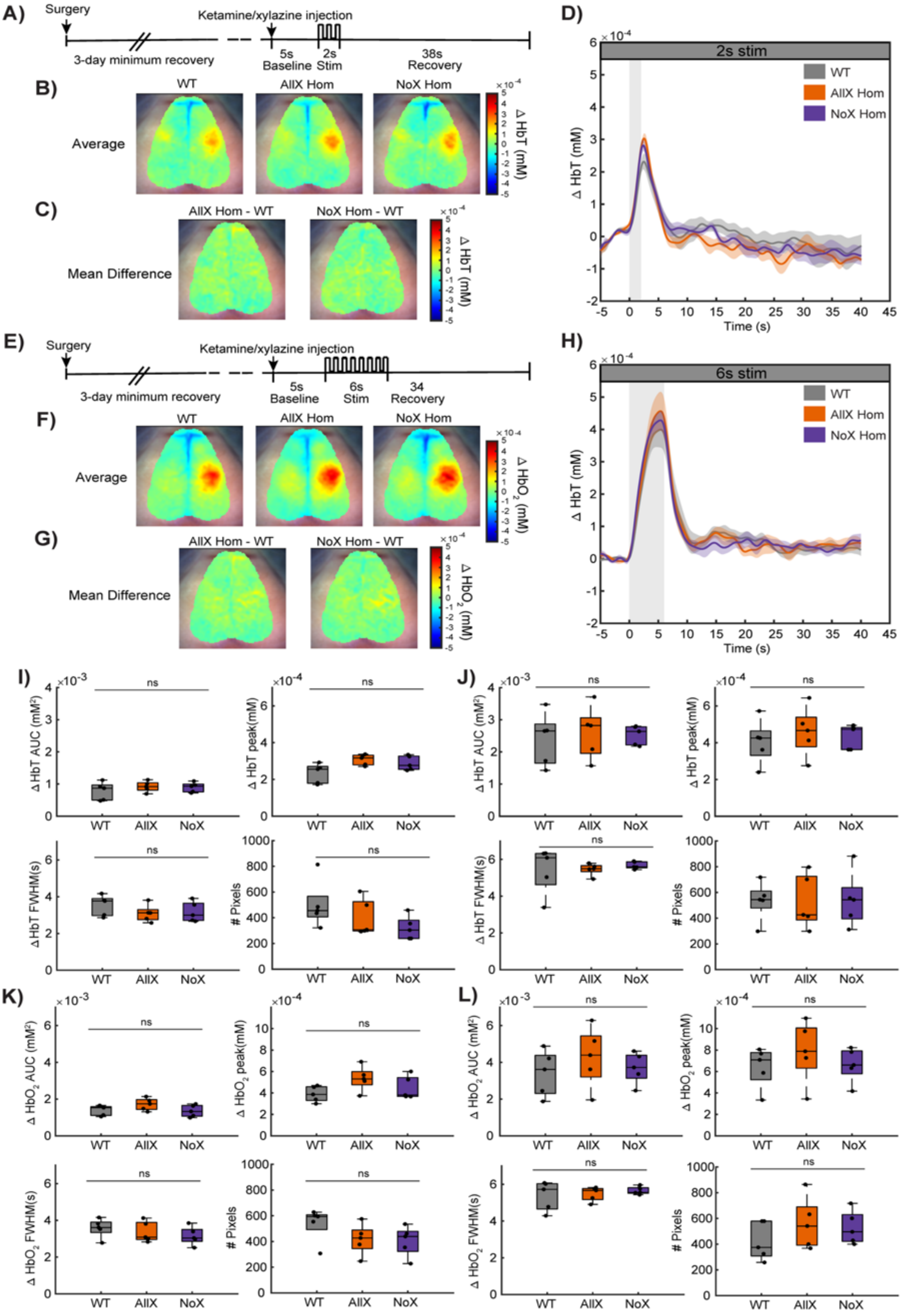

Supplemental Figure 4. Hemodynamic response to somatosensory stimuli is not altered in AIIX^{Hom} or NoX^{Hom} mice. A) Schematic of 2s left forepaw electrical stimulation paradigm. **B)** Mean total hemoglobin (HbT) response maps to 2s forepaw stimulus for WT (n=5 mice, n=15 runs), AIIX^{Hom} (n=5 mice, 14 runs) and NoX^{Hom} (n=5 mice,n=15 runs) mice comparing the time period 1-6s post-stimuli onset to baseline. **C)** Mean difference between AIIX and WT maps (AIIX^{Hom}-WT) and between NoX^{Hom} and WT maps

(NoX^{Hom}-WT). **D)** Mean HbT response time courses for the same groups. Time courses were extracted from pixels that reached ≥50% of the max peak response in each group. *Shading depicts SEM*. **E)** Schematic of 6s left forepaw electrical stimulation paradigm. **F)** Mean HbT response maps to 6s forepaw stimulus for WT (n=5 mice, 25 runs, AllX (n=5 mice, 20 runs) and NoX (n=5 mice, 20 runs) mice comparing the time period 0-6s post-stimuli onset to baseline. **G)** Mean difference between AllX and WT maps ($AllX^{Hom}$-WT) and between NoX and WT maps (NoX^{Hom}-WT). **H)** Mean HbT response time courses for the same groups. Time courses were extracted from pixels that reached ≥50% of the peak response in each group. *Shading depicts SEM* **I)** Analysis of HbT response parameters to 2s stim, including area under the curve (AUC), peak response amplitudes, full-width half-maximum (FWHM), and number of pixels reached ≥50% max response amplitude. **J)** Analysis of HbT response parameters to 6s stim. **K)** Analysis of oxyhemoglobin (HbO_2) response parameters to 2s stim. L) Analysis of HbO_2 response parameters to 6s stim. ns = not significant by one-way ANOVA.
