# Supplementary material for: Evaluation of gliovascular functions of AQP4 readthrough isoforms
Source: Front Cell Neurosci. 2023 Nov 23;17:1272391. doi: 10.3389/fncel.2023.1272391 (PMC10701521; doi:10.3389/fncel.2023.1272391)
Supplement: Supplementary file 8 [file Image_5.pdf]

SUPPLEMENTARY MATERIAL

Evaluation of gliovascular functions of Aqp4 readthrough isoforms

Shayna M. Mueller*[1,2], Kelli McFarland White*[1,2], Stuart B. Fass[1,2], Siyu Chen[1,2,3], Zhan Shi[4], Xia Ge[3,6], John A. Engelbach[3,6], Seana H Gaines[3], Annie R Bice[3], Michael J. Vasek[1,2], Joel R. Garbow[3,6], Joseph P. Culver[3,7,8,9,10], Zila Martinez-Lozada[11], Martine Cohen-Salmon[12], Joseph D. Dougherty[++1,2,6], Darshan Sapkota[++4,5]

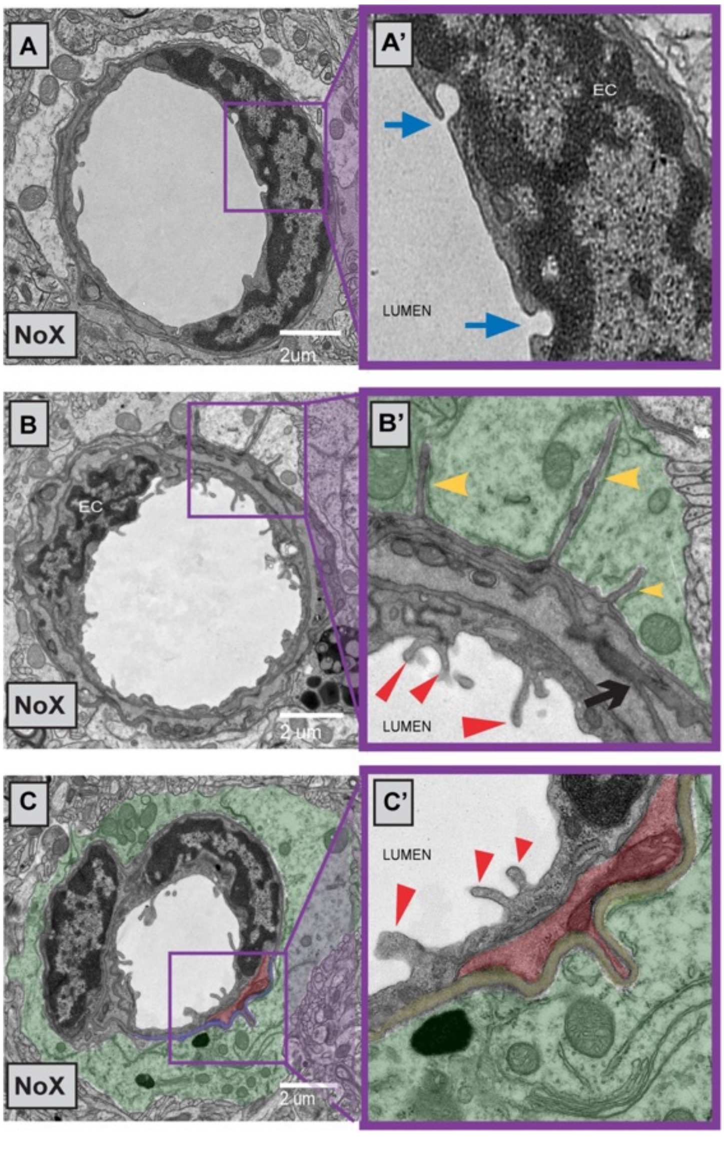

Supplemental Figure 5. Cortical vasculature ultrastructural morphology largely unchanged in AQP4 readthrough mutants. A-C) Representative EM images of NoX blood vessels depicting quantified ultrastructures. **A' inset) Budding** endothelial cell vesicles (blue arrows). **B' inset)** endfoot (light green), basal lamina "projections without contents" (yellow arrowheads), microvilli (red triangles), lamina branching (black arrow). **C' inset)** microvilli (red triangles), endfoot (light green), basal lamina "projection with contents" (red, presumed pericytes).
