# Supplementary material for: Evaluation of gliovascular functions of AQP4 readthrough isoforms
Source: Front Cell Neurosci. 2023 Nov 23;17:1272391. doi: 10.3389/fncel.2023.1272391 (PMC10701521; doi:10.3389/fncel.2023.1272391)
Supplement: Supplementary file 11 [file Image_8.pdf]

SUPPLEMENTARY MATERIAL

Evaluation of gliovascular functions of Aqp4 readthrough isoforms

Shayna M. Mueller*[1,2], Kelli McFarland White*[1,2], Stuart B. Fass[1,2], Siyu Chen[1,2,3], Zhan Shi[4], Xia Ge[3,6], John A. Engelbach[3,6], Seana H Gaines[3], Annie R Bice[3], Michael J. Vasek[1,2], Joel R. Garbow[3,6], Joseph P. Culver[3,7,8,9,10], Zila Martinez-Lozada[11], Martine Cohen-Salmon[12], Joseph D. Dougherty[++1,2,6], Darshan Sapkota[++4,5]

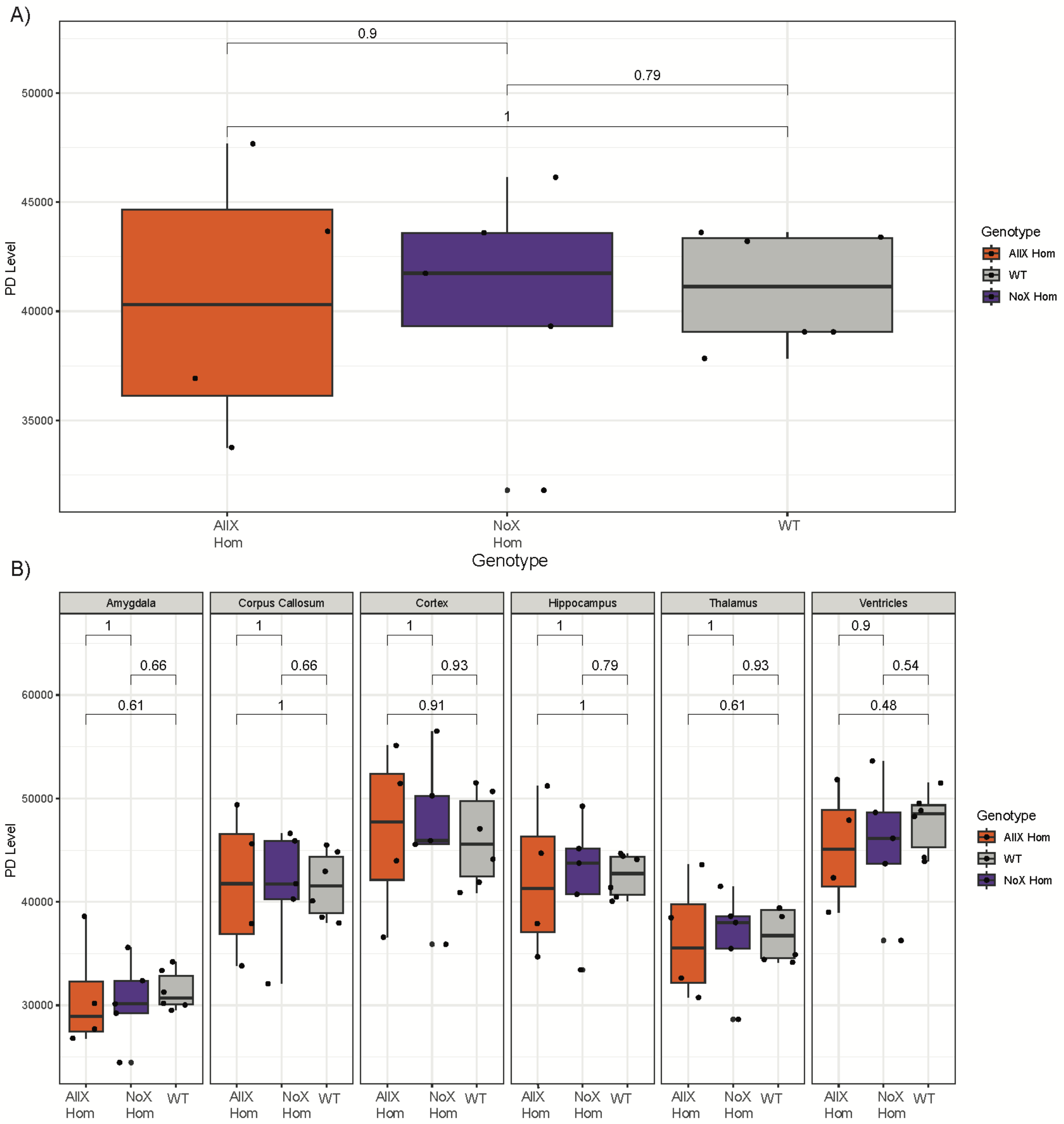

Supplementary Figure 8: Proton density levels as proxy for brain water content shows no significant differences between genotypes. A) Proton density levels for each of AllX Hom, NoX Hom and WT genotypes shows no significant difference in brain water content (mean of 6 brain regions) by Wilcox test between genotypes. **B)** Proton density levels are shown for each brain region separately with no significant differences in genotypes in any brain region by Wilcox test. Each data point shown represents 1 mouse.
